# A survey of people with foot problems related to rheumatoid arthritis and their educational needs

**DOI:** 10.1186/s13047-017-0193-6

**Published:** 2017-03-06

**Authors:** Andrea S. Graham, John Stephenson, Anita E. Williams

**Affiliations:** 1 Centre for Health Science Research, University of Salford, Frederick Road, Salford, UK; 2 Directorate of Prosthetics, Orthotics and Podiatry, University of Salford, Frederick Road, Salford, UK; 3 School of Human and Health Sciences, University of Huddersfield, Queensgate, Huddersfield UK

## Abstract

**Background:**

Up to 50% of people with rheumatoid arthritis (RA) have foot symptoms at diagnosis, hence early foot health intervention is recommended and this should include patient education. This study identifies, for the first time, the foot health education (FHE) needs of people with RA.

**Methods:**

An online survey of people with RA (*n* = 543) captured quantitative data in relation to the aims, methods of delivery, content, timing and accessibility of FHE.

**Results:**

The majority concurred about the aims of FHE. Verbal delivery and websites were the most common methods. Written and verbal FHE were perceived to be the most effective methods. The point of diagnosis was the preferred time to receive it. Lack of access to FHE included minimal focus on foot health during consultations by both health practitioners and patients with RA. Participant gender, age, disease duration and living situation had a statistically significant influence on the results.

**Conclusion:**

Foot health education is rarely considered within the medical consultation. There is a lack of patient and/or health professional awareness of this need with a detrimental impact on foot health. Patients require health professionals to identify their foot education health needs. Tailored foot health education should begin at initial diagnosis.

**Electronic supplementary material:**

The online version of this article (doi:10.1186/s13047-017-0193-6) contains supplementary material, which is available to authorized users.

## Background

Rheumatoid arthritis (RA) has a significant impact on foot-related morbidity [1, 2], with associated physical pathology manifesting in the feet as deformity [3–5], callus and ulceration [6, 7], and both vascular [8] and neurological deficit [9]. Pharmacological management of RA has additional consequences for foot health, with medications being associated with increased risk of infection [10, 11]. The sequelae of this spectrum of foot pathology are loss of function, reductions in mobility, quality of life and social participation [12] and a potential negative impact on self- image [13].

There is a growing body of evidence to support effective management of foot pathology in RA, [14–16], with foot health education (FHE) being recognised as being an essential component, or as an intervention in its own right. However, some people with RA experience sub-optimal foot health with a lack of understanding of the relationship between the disease and foot health or lack of knowledge about the NHS services available to them [17, 18]. Importantly, there is evidence that people with RA do not understand the role of the podiatrist or self-management strategies they might use, in the improvement or maintenance of their own foot health [17–19]. The positive effect of patient education in relation to general disease management and overall health is well recognized in RA [20, 21]. The persistence of sub-optimal foot health in RA can be potentially damaging to overall health [22]. Therefore, improving patient’s knowledge of foot health and management (either self or professional) is considered essential to their overall well being, functional ability and quality of life.

Having an understanding of the FHE needs of people with RA in respect of the content, timing, mode of delivery and potential barriers to its provision, is a crucial step to achieving good foot health in this patient group. Therefore, this study aims to identify, for the first time, what people with RA need in relation to FHE and the barriers to its’ provision.

## Methods

Ethical approval was received from the University of Salford, Research Innovation and Academic Engagement Ethical Approval Panel (HSCR12/35). 

### Questionnaire design

The survey questionnaire was designed to capture quantitative and qualitative data from people with RA from across the UK. Questions were developed from a literature search and the results of previous exploratory work that informed the content of the questionnaire [19, 23]. To ensure face and content validity the survey was piloted with four people with RA, recruited from the University of Salford, Podiatry Clinic. ‘Think aloud’ cognitive debriefing [24, 25] was used in order to reduce sources of response error, ensure clarity of questions and the overall structure of the questions. The results of the pilot led to a small number of changes relating to the question clarity and the consolidation of items within two sections (section 3 and 4) relating to verbal and ‘one-to-one’ methods of delivery.

The main components of the final survey consisted of six sections, with 16 questions in total, including demographic questions (section 1, questions 1–7), including an option for participants to add in additional free-text responses (questions 10, 13–15) if they had responses that were not included within the survey response set and a free-text comment question (question16) for the whole survey (Additional file 1). The results from the qualitative analysis of the free text comments have been published [19].

The score obtained by each participant in each section was obtained by a summation of the individual item scores within each section. Table 1 outlines the score system for each section/question.

**Table 1:** Survey score system by section and question type

Section & Question type/ number	Section title	Section Score system
Section 2; question 8 – Likert 5-point agree/disagree scale.	What the aims of foot health education are	A summed total of item scores relating to: understanding about treatments consented for; informed choices about treatment options; enablers for foot safety; education about the effects of RA; information about available resources.
Section 3, questions 9–11- Q9- Q10- Q11-Likert 3-point importance scale for perceived effectiveness of method	The best ways of receiving foot health education and effectiveness of method	A summed total of item scores relating to various components of methods of delivery: written, verbal and group information; use of audio-visual demonstrations, images and videos; and websites.
Section 4, question 12 – Likert 5-point agree/disagree scale	What should be included in foot health education	A summed total of item scores relating to the participants’ opinions of how important it was to know about each component of FHE content related to RA.
Section 5, question 13: Multiple choice question	When is the best time to receive foot health education	A summed total of item scores relating to participants’ opinions of the best time to receive foot health education/information.
Section 6, questions 14–15 Q14– Likert 5-point agree/disagree scale Q 15 -Multiple choice question	Access to foot health education/information and website use.	A summed total of item scores relating to various components of access: positive statements relating to barriers to access negative statements relating to barriers to access, and commonly accessed websites.

The questionnaire was anonymous, self-administered and of a cross-sectional observational design using a web based survey the Bristol Online Survey website (https://www.onlinesurveys.ac.uk/). A mixture of open-ended, closed-ended dichotomous, contingency, nominal and ordinal polytomous questions were used to reduce the risk of missing data [26, 27].

### Participants

Inclusion criteria were: a diagnosis of RA, patient membership of the National Rheumatoid Arthritis Society (NRAS), ability to understand written English and an ability to access the internet. Participants were recruited through NRAS membership via e-mail invitation with a web-link to the survey. At the time of survey development NRAS membership numbers were 3351, of which approximately 630 were healthcare professionals, giving a potential sample population of 2731. The recruitment period ran from September to November 2013, with potential participants receiving an initial e-mail invite and requesting any members that were health care professionals, spouses or carers not to complete the survey. A ‘reminder’ e-mail was sent after 2 weeks. Consent was implicit by the completion of the survey and participants were informed of this at the start of the online survey.

### Data analysis

Data was analysed using SPSS v 20.0 (SPSS, Chicago, IL, USA). The sample was summarised descriptively. Inferential analyses were conducted. Independent samples *t*-tests were conducted to assess the effect of factors including gender, age (dichotomised into age 59 or younger, age 60 or above) and living situation (dichotomised into living with partner/carer or not living with partner) as appropriate on the component scores which formed the outcome measures of the study.

A *p* value of < 0.05 was considered to indicate statistical significance (Additional file 2).

## Results

Five hundred forty-three people with RA completed the survey. The majority of respondents in this study were female (89.7%, *n* = 487), aged between 40 and 69 years of age (85.5%, *n* = 464) and had disease duration of more than 5 years (67.3%, *n* = 365), with younger participants tending to have shorter disease duration (22.5%, *n* = 122 of participants aged under 59 years had a disease duration of less than 5 years, compared with 10.3%, *n* = 56 participants aged over 60 years), though this result could be said to be implicit.

There was a wide geographical spread of participant representation across the UK. Access to foot health services is patchy across England and the non-English regions, with the South East, North West and South West of England showing the largest percentage of respondents to access podiatry (Table 2).

**Table 2:** Results from survey question 5: the number of participants receiving podiatry, cross-referenced with participants region of residence

Main UK region of residence	Frequency (%) of respondents in each region receiving podiatric treatment
South East England	46 (38.7%)
North West England	27 (45.0%)
South West England	30 (40.0%)
Greater London	10 (25.0%)
West Midlands	10 (25.6%)
East Anglia	7 (18.4%)
Yorkshire and North Humber	15 (44.1%)
East Midlands	16 (50.0%)
South Central England	10 (52.6%)
North East England	16 (51.6%)
Wales	6 (37.5%)
Scotland	20 (58.8%)
Northern Ireland	4 (67.3%)

These results remain similar when both NHS and Private Practice podiatry provision are identified (Table 3). Lack of access to podiatry services could be a potential barrier to people with RA receiving FHE, with only 33.7% (*n* = 183) of the participants stating that they had received FHE.

**Table 3:** Results from survey question 6: number of participants receiving either private or NHS podiatry, cross-referenced with participants region of residence

Main UK region of residence	Frequency (%) of respondents in each region who receive podiatric treatment receiving podiatric treatment from:		
	NHS ONLY	Private practice ONLY	Both NHS and private practice
South East England	30 (61.2%)	14 (28.6%)	5 (10.2%)
North West England	18 (69.2%)	4 (15.4%)	4 (15.4%)
South West England	22 (71.0%)	8 (25.8%)	1 (3.2%)
Greater London	6 (50.0%)	5 (41.7%)	1 (8.3%)
West Midlands	5 (50.0%)	3 (30.0%)	2 (20.0%)
East Anglia	2 (25.0%)	6 (75.0%)	0 (0.0%)
Yorkshire and North Humber	8 (53.3%)	4 (26.7%)	3 (20.0%)
East Midlands	10 (55.6%)	8 (44.4%)	0 (0.0%)
South Central England	7 (70.0%)	3 (30.0%)	0 (0.0%)
North East England	11 (68.8%)	3 (18.8%)	2 (12.5%)
Wales	5 (71.4%)	2 (28.6%)	0 (0.0%)
Scotland	14 (70.0%)	2 (10.0%)	4 (20.0%)
Northern Ireland	2 (50.0%)	2 (50.0%)	0 (0.0%)

### Aims of Foot health Education

Over 80% of the participants agreed with all the aims of foot health education (Table 4), with between 4 and 10% disagreeing and 10% ‘didn’t know’.

**Table 4:** Survey Items in relation to the aims of Foot Health Education

Survey items in relation to the Aims of FHE:
• So I understand about the treatments I give consent for
• To allow me to make informed choices about my treatment options
• To enable me to look after my own foot health safely
• To educate me about how RA can affect my feet
• To inform me about information resources I can access such as; websites or support groups.

The age of the participants (dichotomised into under-60 versus 60 or over) was substantively related to the FHE-Aims score; with mean scores of 9.04 in the under 60s group and 8.42 in the 60-and over group. The difference of 0.62 units approached statistical significance (*p* = 0.073) using an independent samples *t*-test. The effect was small in magnitude as measured by Cohen’s *d* statistic (*d* = 0.154).

### The best ways of receiving foot health education

66.3% of participants had never received information or education about their feet or how to care for them because of RA. For the remaining 33.7%, the most common methods of delivery were; verbal information provided by the Podiatrist (26.3% of the total sample) and other Allied Health Professionals (AHPs) (31.5% of the total sample) and via signposting to websites (23% of the total sample), a method mostly used by Specialist Nurses and other AHP’s (3.3% by podiatrists). Only 81 participants (15%) stated that they had received written information from any profession. Other methods of delivery such as Group Education sessions and the use of audio-visual aids such as DVD’s, self-care demonstrations or the specific use of images to aid educational delivery were infrequently accessed.

The living situation of participants (whether they live alone or with a significant other) had a statistically significant effect on the methods of FHE provision experienced by the participants. Specifically, an independent samples *t*-test revealed that there was a statistically significant relationship between the provision of written information (*p* = 0.008) and the living situation of the participants with those that lived with a significant other being more likely to receive written information than those who lived alone.

When asked how effective the methods of delivery were perceived to be, written and verbal provision were ranked the highest by over 75% of participants. Website-based information was 3^rd^ highest, with 70% of participants perceiving this to be an effective mode of delivery.

### What should be included in foot health education?

80–93% of the participants considered that information on how RA can affect the feet, how RA-related medication can affect the feet, and what might happen if they didn’t look after their own feet as ‘very important’. Information about the role of the podiatrist, foot health interventions and how to look after their own feet were also considered very important by 73–79% of participants. Over half (51–68%), considered that general disease related information, contact details for AHPs, how other AHPs are involved with foot health and information relating to patients support groups/website resources, as being very important.

An independent samples *t*-test revealed that there was a statistically significant relationship between the genders of the participants for main effects size in relation to FHE content (*p* = 0.022). The effect was medium in magnitude as measured by Cohen’s *d* statistic (*d* = 0.326). Female participants were more likely to consider the inclusion of information on the role of the podiatrist, information about RA medication and its’ effect on the feet, contact details and information about treatment options, as very important.

### When is the best time to receive foot health education?

Participants were asked when they thought would be the best time to receive foot health education relating to RA. The most popular time for receiving foot health education was considered to be at the point of diagnosis, by 78% of the respondents, with only 36% agreeing that foot health education should only be provided when they asked for it. The association between gender and the timing of FHE achieved statistical significance at the 5% significance level using an independent samples *t*-test (*p* = 0.019) with female participants, who agreed to 2.22 statements on average (SD 1.06) about when FHE should be provided, being more likely to agree with the statements than male participants (who agreed with 1.88 statements on average (SD 0.98). The effect was medium in magnitude as measured by Cohen’s *d* statistic (*d* = 0.332). In particular, female participants appeared more likely to agree that it should be provided on demand.

### Access to foot health education/information

When asked about factors relating to their ability to access and opportunities for accessing foot health information or education, 46% were not clear about what they should ask AHPs in relation to their foot health and RA. 62% of participants had not been asked about their foot health during their appointments with other AHPs, although 53 people provided additional comments to say that they had initiated a dialogue about their feet with the AHP.

71.5% had not received written foot health information from either their podiatrist or other AHP. However, 64% knew where they could access written information in relation to foot health, either as a leaflet format or through the internet. The majority of participants (92.5%) were able to easily access the internet. Over a third had been able to find information but they had found some difficulty in understanding the information. Time and finances were not a barrier to attending meetings where education could be provided (60 and 71% respectively).

The age of the participants had a statistically significant relationship in relation to their perception of barriers to FHE provision (positive items) (*p* = 0.004) where participants who were less than 59 years of age were more likely to disagree or strongly disagree with the item ‘I am clear about what questions to ask my podiatrists or other Health Professional about my feet’ and to enter a ‘don’t know’ response to the items. The effect was small in magnitude as measured by the ϕ statistic (ϕ = 0.179).

The most commonly accessed website for foot health information was NRAS at 76.4%, Arthritis Research UK (32.2%) and Arthritis Care (27.6%) with 11–12.7% using WebMD and patient.co.uk. The gender of the participants would achieve statistical significance at the 5% significance level in relation to the website of choice (*p* = 0.004) with a greater ratio of female participants more likely to use the NRAS website. The effect was small in magnitude as measured by the ϕ statistic (ϕ = 0.122).

## Discussion

The aim of this study was to identify what people with RA need in relation to FHE and the potential barriers to its’ provision. This is the first study to describe the current provision of foot health education (FHE) to people with RA across the UK. It has identified the lack of access that many people with RA experience in relation to foot health services and this being a significant factor in accessing RA-related information and resources. The participants were very clear in what they required and desired in relation to FHE.

Forty percent (*n* = 217) of respondents stated that they received podiatry treatment, of which only 162 people receive NHS podiatry. This could reflect a lack of participant awareness of foot health service provision [18] and the geographical skew of a higher proportion of podiatry service provision for people with rheumatic diseases within certain areas of the United Kingdom, such as South Central England, North East England, Scotland and Northern Ireland with over 50% of sample participants from each of these regions were receiving podiatric treatment [28]. However, the largest numbers of participants receiving podiatric treatment were found as expected in the regions of higher population, particularly higher populations of elderly people, including the South West, South East and North West regions of England. This apparent ‘post-code’ lottery of foot health service provision across the UK means that many people with RA are denied access to those health professionals who are best placed to provide effective and timely FHE. However, poor access to rheumatology-related foot health services is by no means limited to the UK with similar issues relating to timely access to podiatric care identified in Australia [29, 30]. A lack of specialist podiatry services means that it is essential that FHE is provided in a way that is high profile, easily accessible and supports self-management for people with RA-related foot health problems.

The majority of the participants stated that they agreed with the aims of FHE, despite only one third of participants reporting that they had received FHE. Participants who gave a ‘don’t know’ response to the items in this part of the survey were more likely to be under the age of 59 years. This may be because younger participants tended to have shorter disease duration (<5 years) and therefore their educational needs were possibly not as defined. Alternatively, younger participants may have fewer foot symptoms, and hence less physical awareness of the impact that RA can potentially have on their foot health [18]. Further to this, the number of participants in the <59 years age group who had not received FHE (*n* = 198) was greater than those in the >60 years age group (*n* = 161) which could also have reduced their awareness of RA foot-related problems. Additionally, when asked if they; ‘were clear about what to ask my podiatrist or other AHP regarding my foot health’, participants in the younger age group were significantly (*p =* 0.004) more likely to either ‘not know’ or ‘disagree’ with the statement. The effect was small in magnitude as measured by the ϕ statistic (ϕ = 0.179).

The influence of age and disease duration on educational needs in patients with rheumatic disease has been identified by Dragoi et al., [31] who found that older patients with a longer disease duration expressed higher educational needs in relation to pain and movement. Some of the comparisons shown to be significant at the 5% level may not be considered significant in the context of a single finding from multiple testing with a Bonferroni correction applied to correct for multiple comparisons.

Participants rated the importance of the content of FHE as high overall, which supports the value that people with RA place on managing their foot health [18, 19]. Items about the impact that RA and its related medications have on the feet, the role of podiatrist, and the interventions that are used in foot health management and self-management rated particularly highly, showing synergy with the findings of a survey of practitioners’ perceptions of FHE [18]. Female participants rated certain items higher than males: the role of the podiatrist, information about RA medication and its effect on the feet, contact details and information about treatment options were more likely to be rated as very important by female participants. The phenomena of gender influence on educational need or engagement with information-seeking behaviour has been previously identified, with women expressing a higher educational need [31]. Further, they are engaged more in information seeking, positive health behaviours and demonstrating self-efficacy than males [32].

People with RA may benefit from self-managed foot care, providing that it is personalized and the individuals’ physical capability to undertake self-care is assessed [33]. This can help tailor the educational needs of the person in relation to ‘hands-on’ skill. However, their ‘information-needs’ also require recognition and personalization. This ‘needs analysis’ should take into account the potentially differing information needs and skills of those with early or established disease, and the age and gender of the individual concerned. This approach to the identification of educational needs has been shown to be successful in people with RA from a general context, both in the UK and other countries in Europe [22, 34, 35]. The development of a specific foot health educational needs tool could enable people with RA to identify and prioritise their educational needs in a way that is timely and prescriptive to their individual requirements.

The participants reported that they should receive FHE at the time of their diagnosis. The participants’ requirement for early provision of foot health information is supported by that of practitioners [36] and by findings from the qualitative analysis of the participants’ responses [18]. Caution should be exercised so that individuals are not overwhelmed with too much information at the point of diagnosis, although people view access to FHE earlier in the disease with RA as an enabler of self-management and as a way to potentially limit deterioration of foot health [18].

Despite the fact that people with RA *and* practitioners recognize the need for FHE at diagnosis, for many participants there were significant barriers in accessing it. A lack of access to foot health services and poor awareness of how RA can impact on foot health potentially inhibits individuals’ ability to understand what questions they should be asking health professionals about their feet. Being invited to articulate their foot health needs during the consultation is very important for people with RA to allow them to open a dialogue about their feet. However, this opportunity appears to be limited by time, the needs and assessment practices of the consulting practitioner in fulfilling their own clinical ‘agenda’ and the practitioners’ awareness of foot health problems related to RA [18]. Many participants in this study reported that they were not asked about their foot health either during the consultation with their podiatrist (*n* = 113) or with another AHP (*n* = 337), so this opportunity was lost.

Not only did participants lose the chance to engage in verbal FHE, a large number of participants did not receive written information either. Participants reported the provision of written information by any AHP or Rheumatologist to be low (13.6%, *n* = 74); this figure may be compared to a concurrent study of podiatrists, of which 69% (*n* = 29) stated they did provide written information [36]. Written material is a method of delivering information to people that is considered useful once they have left the clinical setting [37] and in increasing knowledge in the short term [38]. People with RA and related foot problems require written information in order to support verbal information provided during the consultation [19].

The most common methods of delivery for FHE were verbal information and sign posting to RA or arthritis-related websites. Although access to FHE can be seen to have been limited at the point of consultation, almost all the participants were able to access the Internet and use it for seeking foot health information from patient support group websites, such as NRAS. The NRAS website was the most likely to be used by participants in this study, although as participants were recruited via NRAS membership, this result is not surprising. Female participants were more likely to use web-based information than men, although the reasons for this are not clear. It may be that females are more motivated to self-care and seek information [32] and the impact of foot-related pathology has more of an impact on their self-image [39].

This study may be perceived to have limitations. Whilst this research reports the perspectives of people with RA in relation to FHE provision, it is limited to the views of people who were recruited through a UK patient support group (NRAS) who also had access to the internet. However, there is no evidence for any systematic differences between such patients and the wider RA population; hence no impact on the generalizability of results is expected. Further, the nature of the sample population and a number of questions within the online survey may mean that the data is subject to response and recall bias [40].

## Conclusion

This study has provided the first insight into the current status of FHE for people with RA in the UK. It has shown that the ‘patchy’ geographical provision of foot health services to this group of people remains similar to that of 10 years ago. Of concern is that people with RA lack awareness of the implications of foot health problems, lack knowledge of where to access information on safe self-management and where and when to access professional foot health services. Patients should be asked about foot health and FHE needs at their medical consultation and signposted to the appropriate service and educational resources if we are to improve foot health and subsequently overall health and quality of life.

The most appropriate time to provide FHE is at initial diagnosis of the disease. FHE needs should be identified and tailored to the individual requirements of the person with RA. Assessment of FHE needs should be undertaken regularly during review appointments. This can be carried out by any health professional that has contact with the patient, not just the podiatrist. In this way foot health information can be provided, or the individual can be signposted to it, in a timely and efficient manner that aligns with the ethos of ‘Making Every Contact Count’ [41].

Verbal information should be offered and supported with written resources, either through the use of leaflets or via appropriate internet-based resources, such as NRAS or Arthritis Research UK.

An information-needs analysis tool should be developed in order to provide an individual with RA the opportunity to articulate their foot health education needs in a way that is personalized, timely and time efficient for their health practitioner. Once this is achieved, an evaluation of FHE will determine how it influences both clinical management and patient outcomes.

Patient education should not be viewed as an adjunct to treatment. Patient education should be at the start and the end of every episode of care and become the mesh through which ‘hands-on interventions’ are connected.

## Additional files

Additional file 1: Rheumatoid Arthritis foot health education survey for patients (PDF 148 kb)
Additional file 2:
*P*-values arising from statistical analyses of participants’ survey responses by section, in relation to gender, age, disease duration and living situation. * - Denotes significance at the 5% level. (DOCX 80 kb)

## Abbreviations

AHP: Allied Health Professional
FHE: Foot Health Education
NHS: National Health Service
NRAS: National Rheumatoid Arthritis Society
RA: Rheumatoid Arthritis
