# Supplementary material for: A survey of people with foot problems related to rheumatoid arthritis and their educational needs
Source: J Foot Ankle Res. 2017 Mar 6;10:12. doi: 10.1186/s13047-017-0193-6 (PMC5340002; doi:10.1186/s13047-017-0193-6)
Supplement: Additional file 1: — Rheumatoid Arthritis foot health education survey for patients (PDF 148 kb) [file 13047_2017_193_MOESM1_ESM.pdf]

Rheumatoid Arthritis foot health education survey for patients

Showing 543 of 543 responses

Showing **all** responses

Showing **all** questions

Section 1

1 Are you

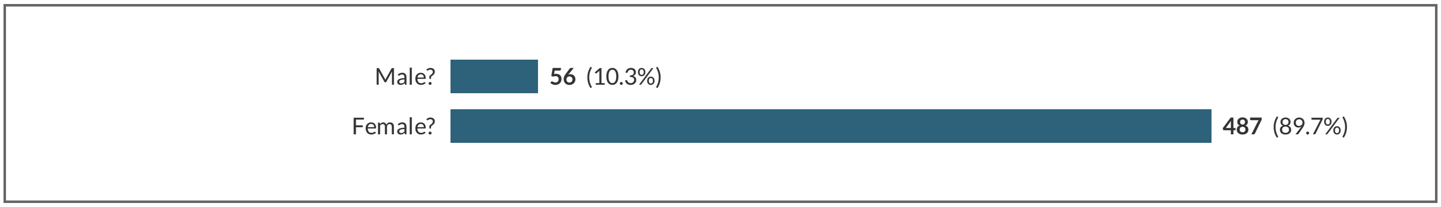

2 What is your current age?From the options below please select the age range that your birth date falls within.

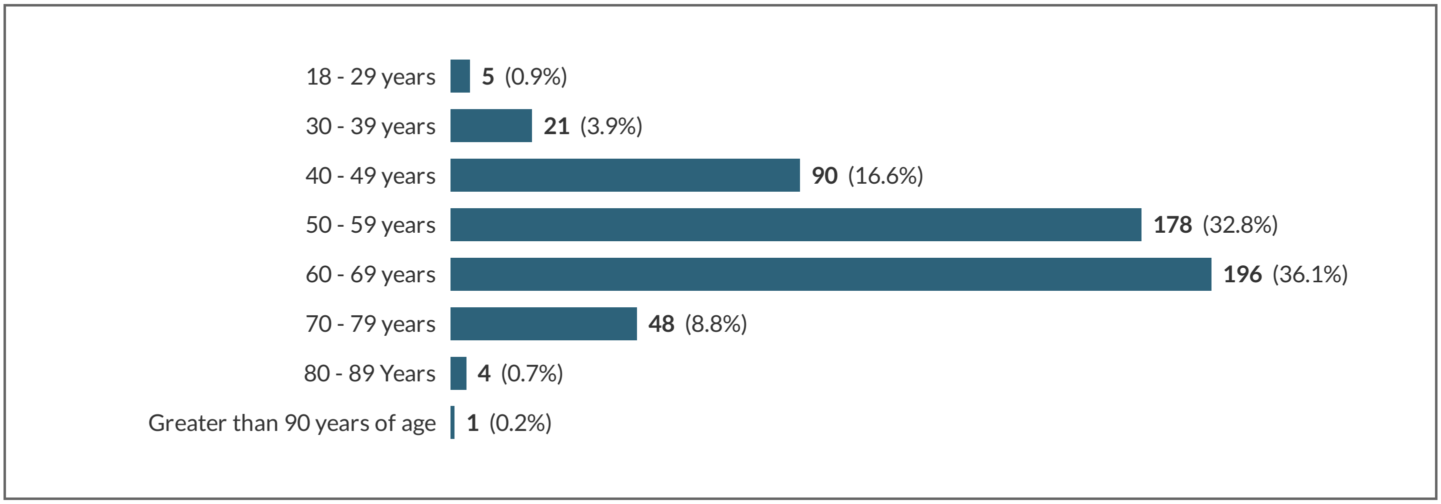

3 Disease duration. Please tell us how long you have been diagnosed with RA by selecting from the options below.

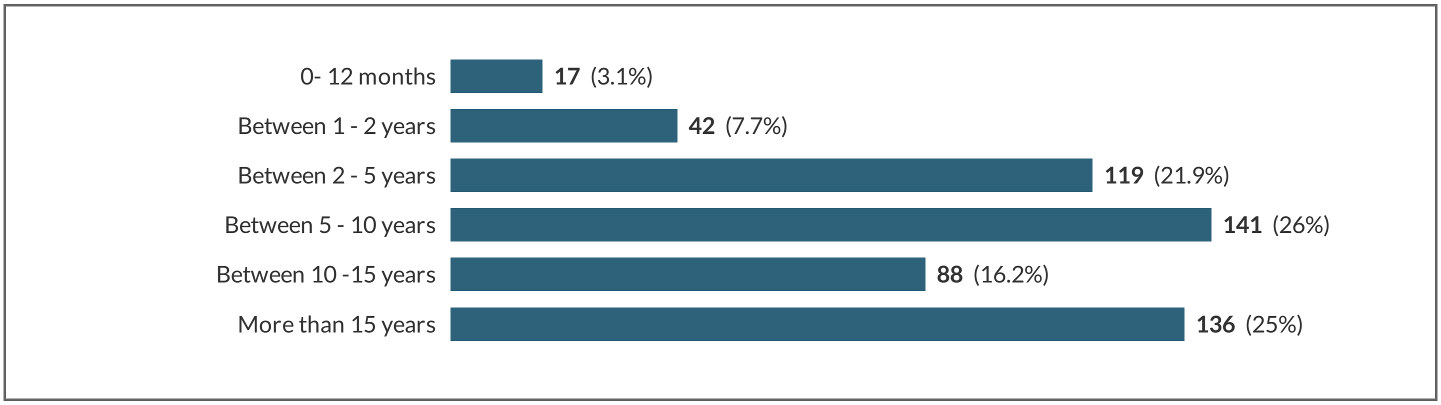

4 Do you live?

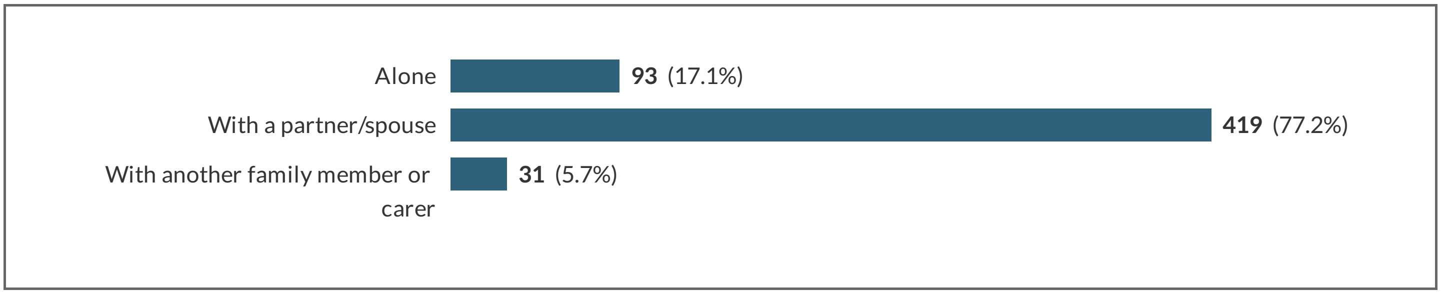

5 Do you currently receive podiatry/chiropody treatment?

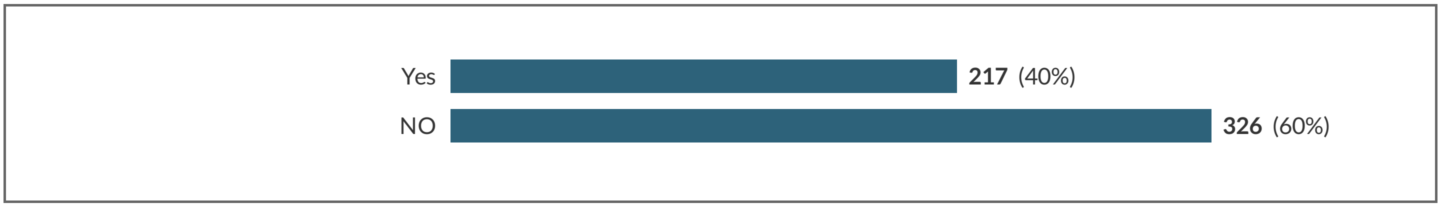

6 If you answered YES to question 5, does your podiatrist/chiropodist work in the?

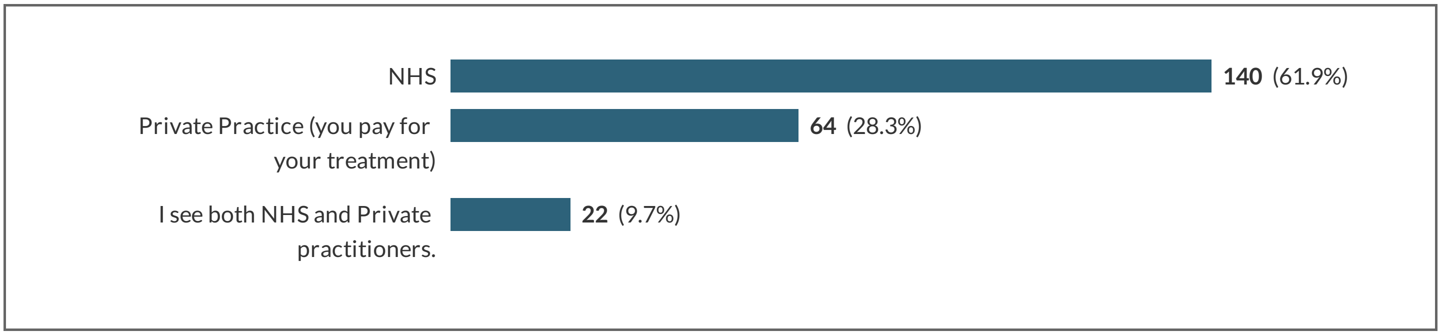

7 Which part of the UK do you mainly live in? (select ONE area only)

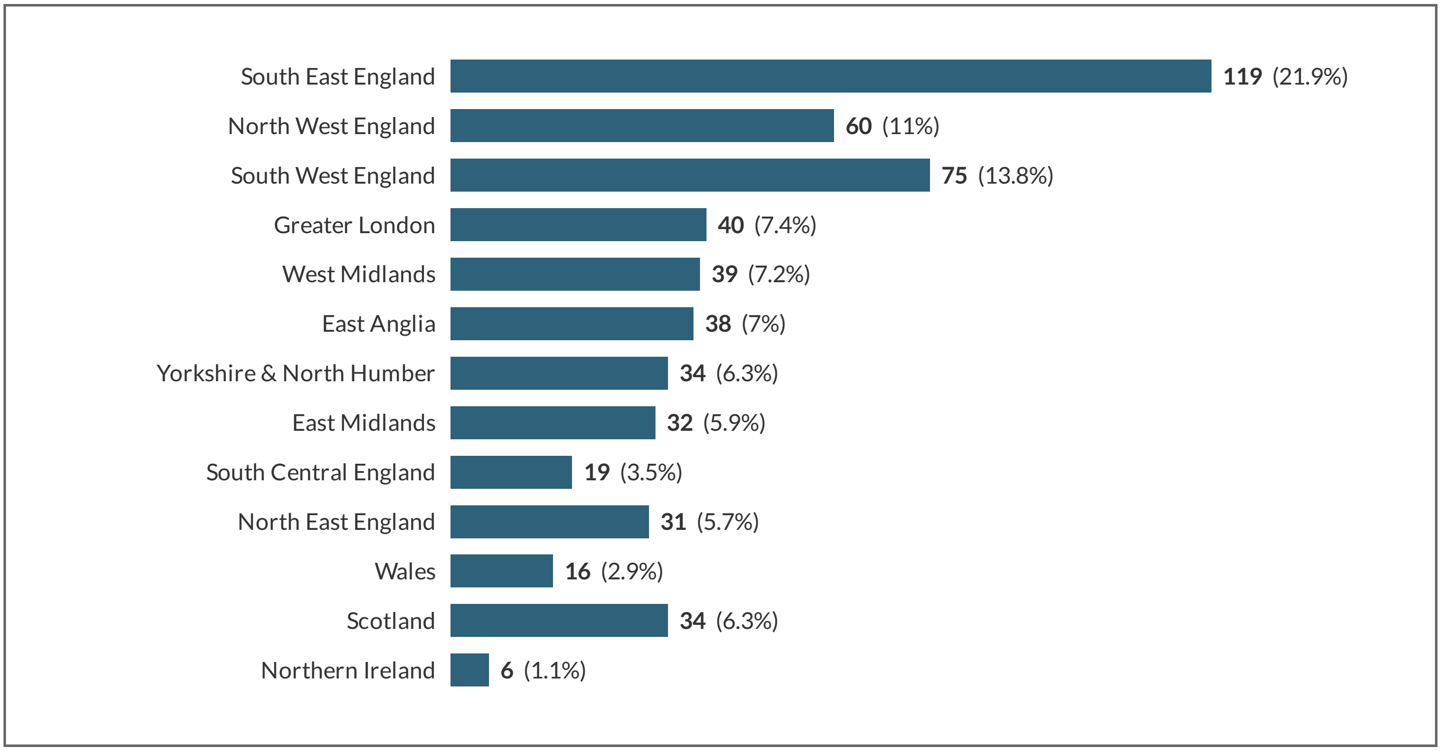

Section 2: The aims of foot health education.

8 To what extent do you agree with the following statements about the AIMS of foot health education.Please select your responses below

8.1 So I understand about the treatments I give consent for

8.1.a So I understand about the treatments I give consent for - Select the extent to which you agree

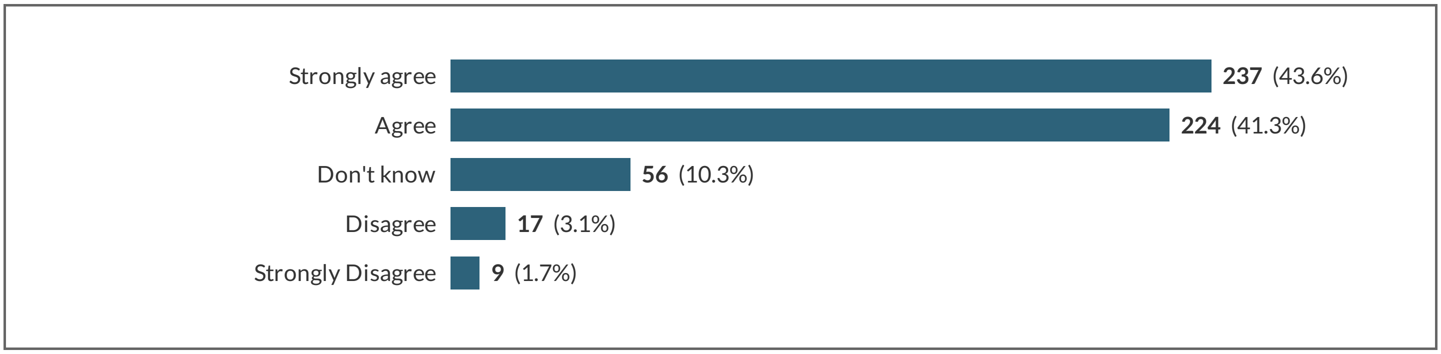

8.2 To allow me to make informed choices about my treatment options

8.2.a To allow me to make informed choices about my treatment options - Select the extent to which you agree

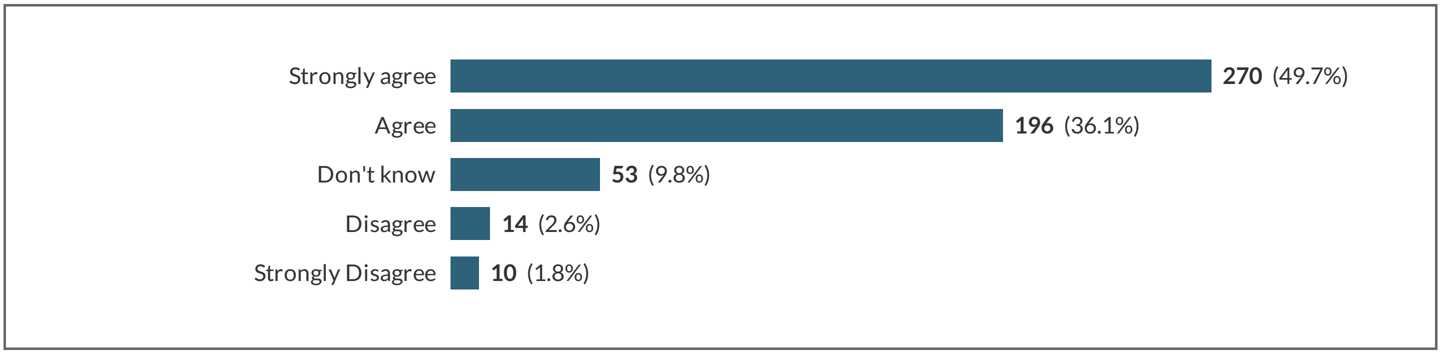

8.3 To enable me to look after my own foot health safely

8.3.a To enable me to look after my own foot health safely - Select the extent to which you agree

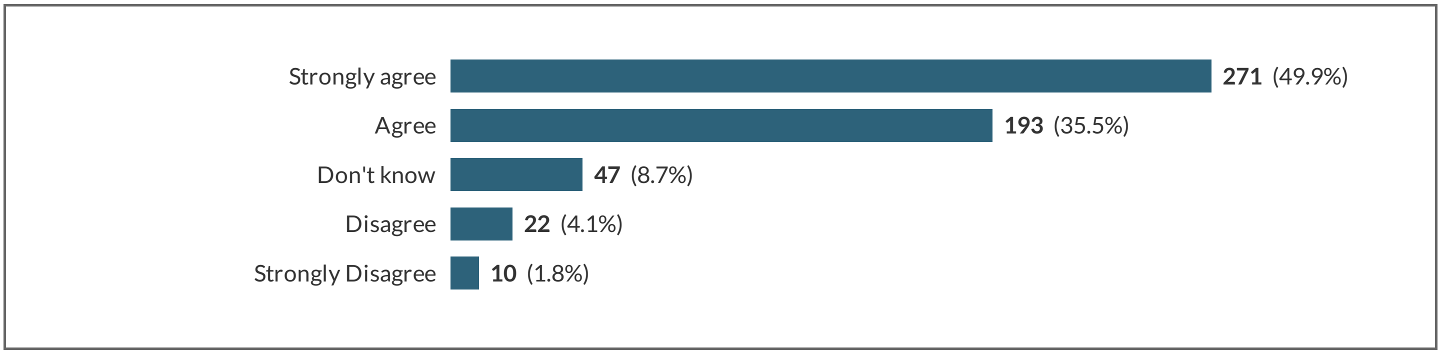

8.4 To educate me about how RA can affect my feet

8.4.a To educate me about how RA can affect my feet - Select the extent to which you agree

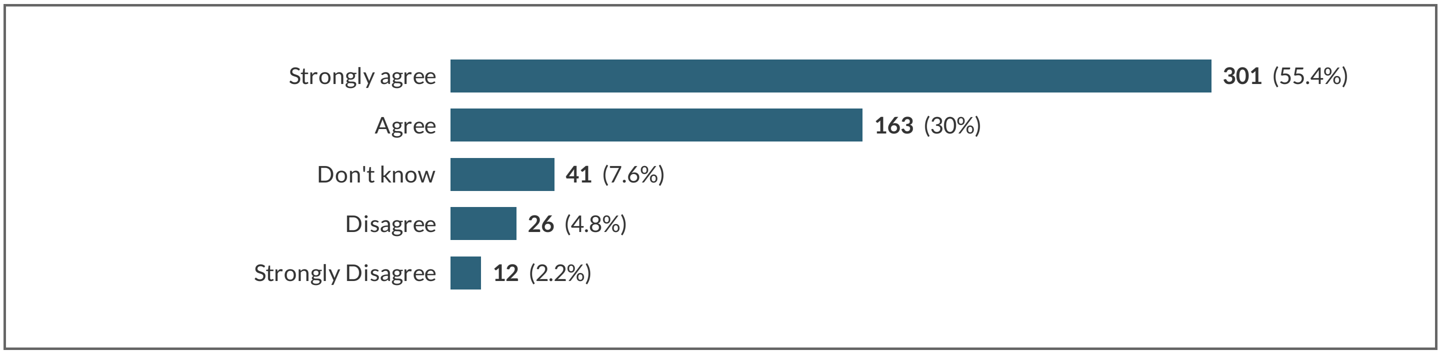

8.5 To inform me about information resources I can access such as websites or support groups (e.g. NRAS, Arthritis Care, Arthritis Research UK)

8.5.a To inform me about information resources I can access such as websites or support groups (e.g. NRAS, Arthritis Care, Arthritis Research UK) - Select the extent to which you agree

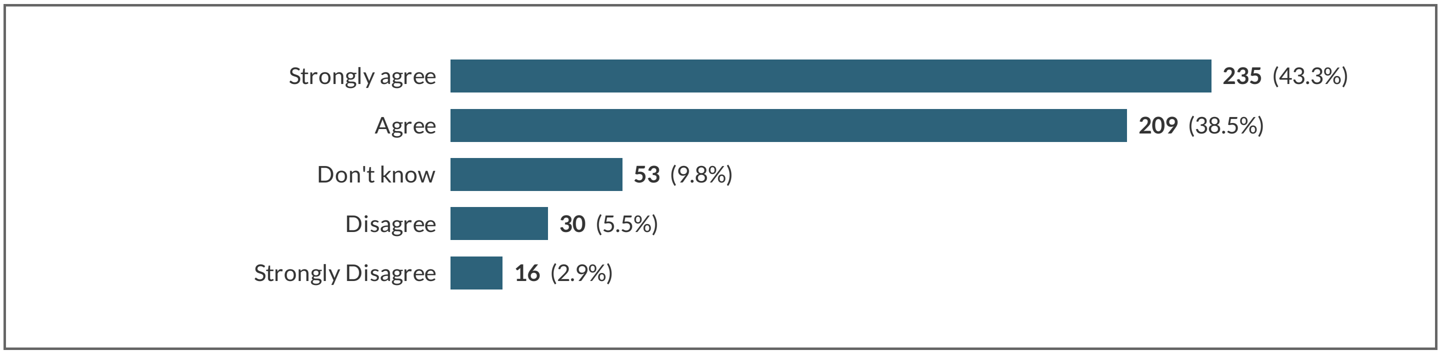

Section 3: The best ways of providing foot health education

9 Have you ever received education or information about how to care for your feet because of your RA?

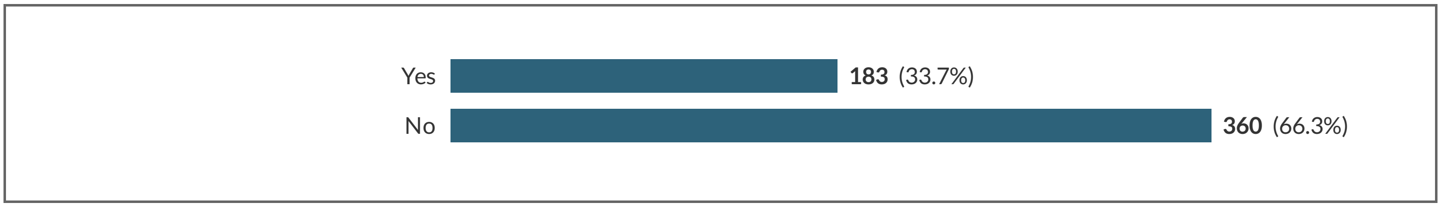

10 If you answered YES' to question 9 and have received any form of foot health information/education, please indicate which types and from whom via the tick box below. Tick all that apply.

10.1 Written Information

10.1.a Written Information - Tick the boxes for each type of education you have received and indicate who you received it from according to the options in the right hand columns.

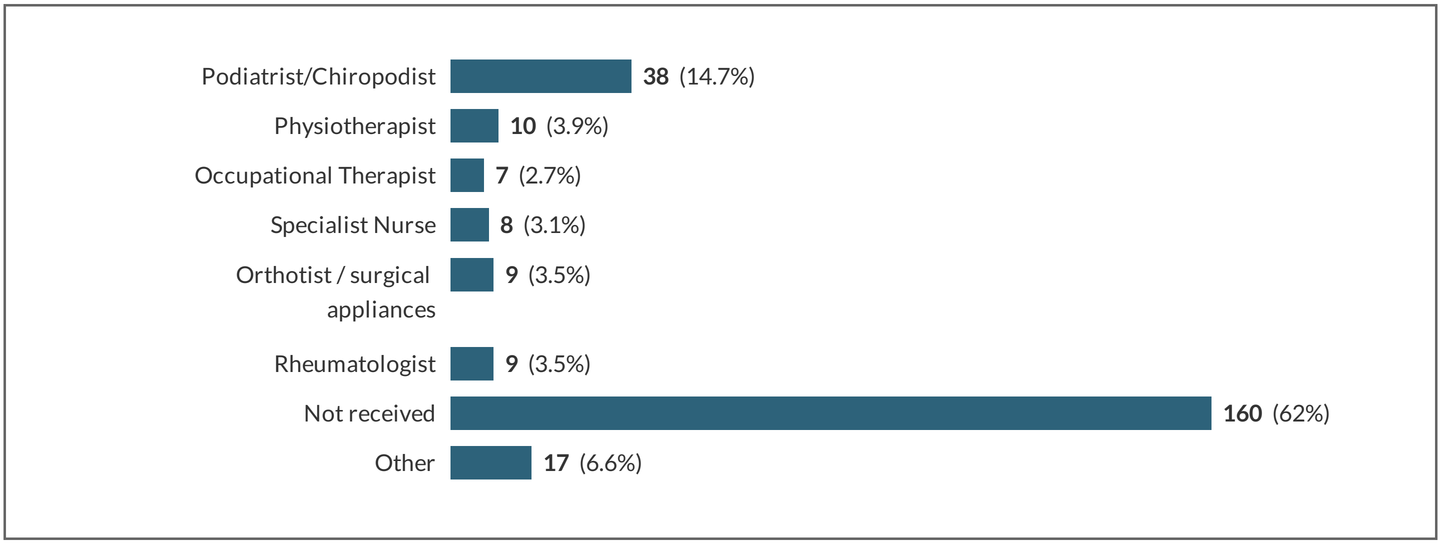

10.1.b Written Information - If you selected Other, please specify:

Showing 5 of 17 responses	
picked the leaflet up myself from hospital	120841-120835-6761313
Arthritis charity website	120841-120835-6761354
Internet	120841-120835-6761450
leaflet	120841-120835-6761655
I downloaded from NRAS site	120841-120835-6761668

10.2 Verbal Information

10.2.a Verbal Information - Tick the boxes for each type of education you have received and indicate who you received it from according to the options in the right hand columns.

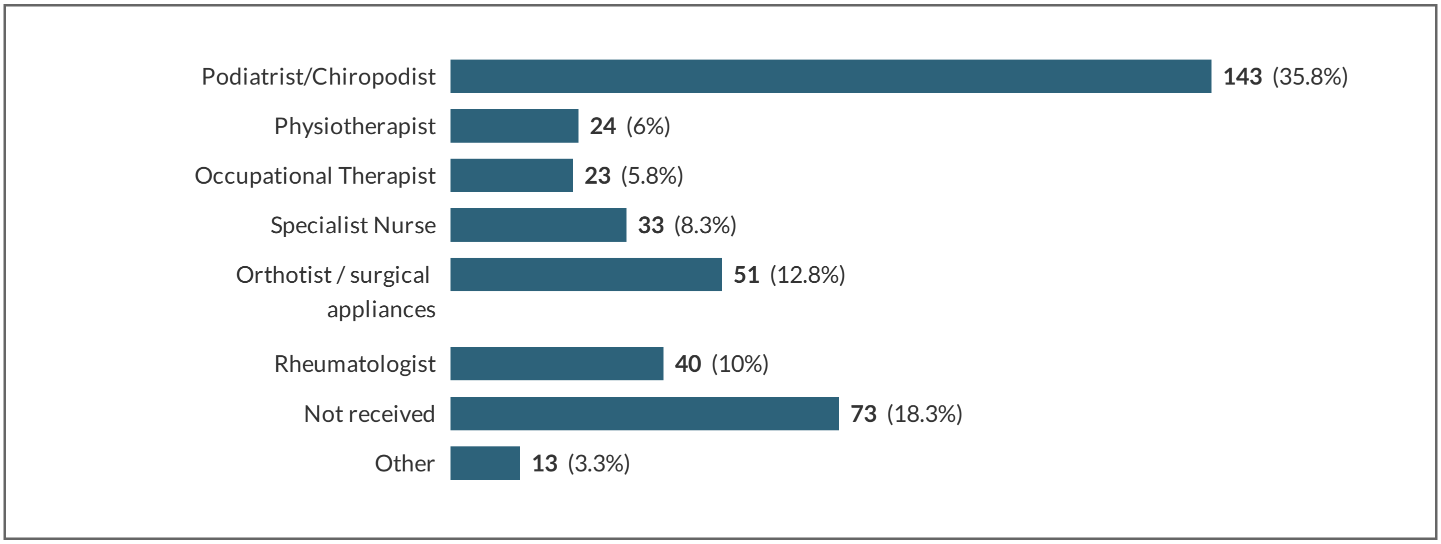

10.2.b Verbal Information - If you selected Other, please specify:

Showing 5 of 13 responses	
foot and ankle orthopaedic surgeon	120841-120835-6761305
podiatrist	120841-120835-6761370
NRAS group	120841-120835-6761425
East Dorset NRAS group	120841-120835-6761488
PRIVATE	120841-120835-6761579

10.3 Group Education sessions

10.3.a Group Education sessions - Tick the boxes for each type of education you have received and indicate who you received it from according to the options in the right hand columns.

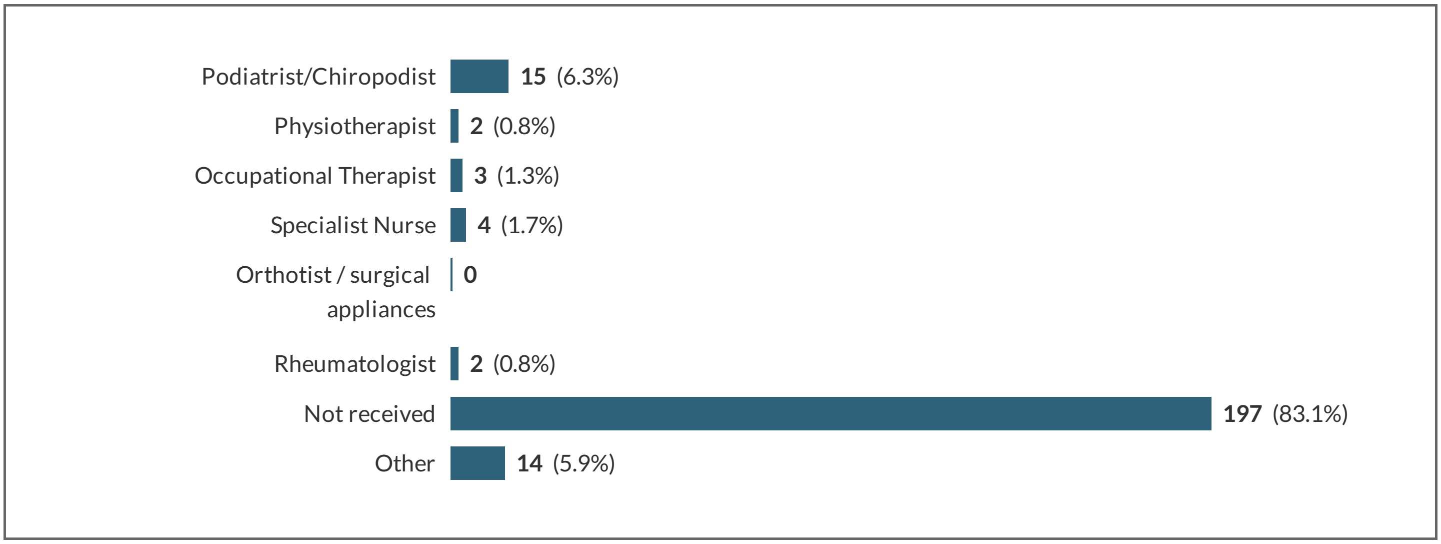

10.3.b Group Education sessions - If you selected Other, please specify:

**Showing 5 of 14 responses**	
trained reflexologist	120841-120835-6761318
NRAS meeting	120841-120835-6761434
NRAS group	120841-120835-6761425
NRAS info day about footcare	120841-120835-6761454
East Dorset NRAS group	120841-120835-6761488

10.4 Audiovisual aids such as short videos or DVD's

10.4.a Audiovisual aids such as short videos or DVD's - Tick the boxes for each type of education you have received and indicate who you received it from according to the options in the right hand columns.

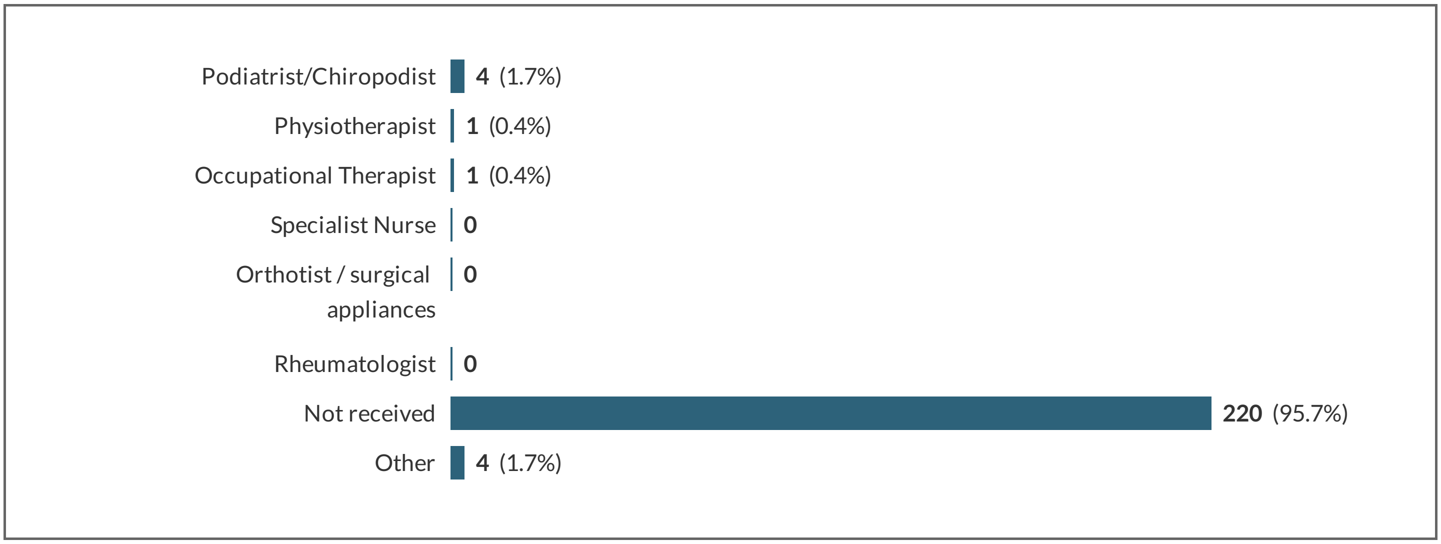

10.4.b Audiovisual aids such as short videos or DVD's - If you selected Other, please specify:

Showing all 4 responses	
Yes from American arthritis website	120841-120835-6761354
Internet	120841-120835-6761450
Web site on own PC	120841-120835-6761738
can't find/remember	120841-120835-6761764

10.5 Audiovisual aids such as demonstrations of how to care for your feet

10.5.a Audiovisual aids such as demonstrations of how to care for your feet - Tick the boxes for each type of education you have received and indicate who you received it from according to the options in the right hand columns.

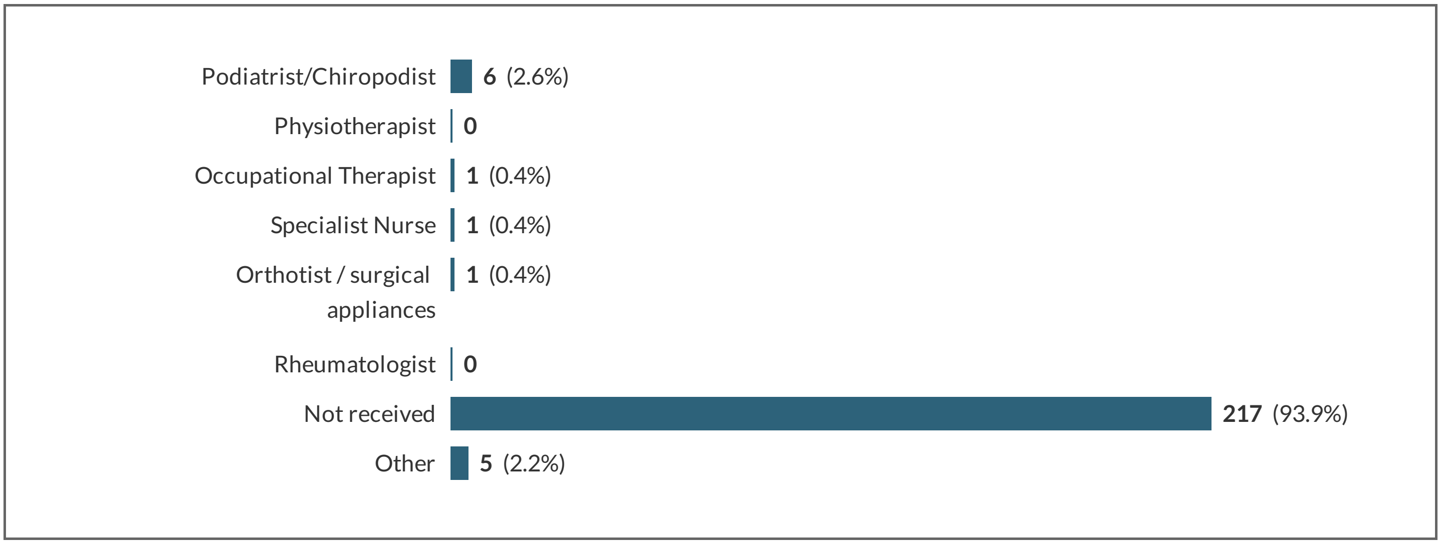

10.5.b Audiovisual aids such as demonstrations of how to care for your feet - If you selected Other, please specify:

Showing all 5 responses	
NRAS meetings	120841-120835-6761434
Internet	120841-120835-6761450
NRAS info day about footcare	120841-120835-6761454
as above	120841-120835-6761738
as above	120841-120835-6761764

10.6 Audiovisual aids such as pictures of footwear/insoles or images of feet

10.6.a Audiovisual aids such as pictures of footwear/insoles or images of feet - Tick the boxes for each type of education you have received and indicate who you received it from according to the options in the right hand columns.

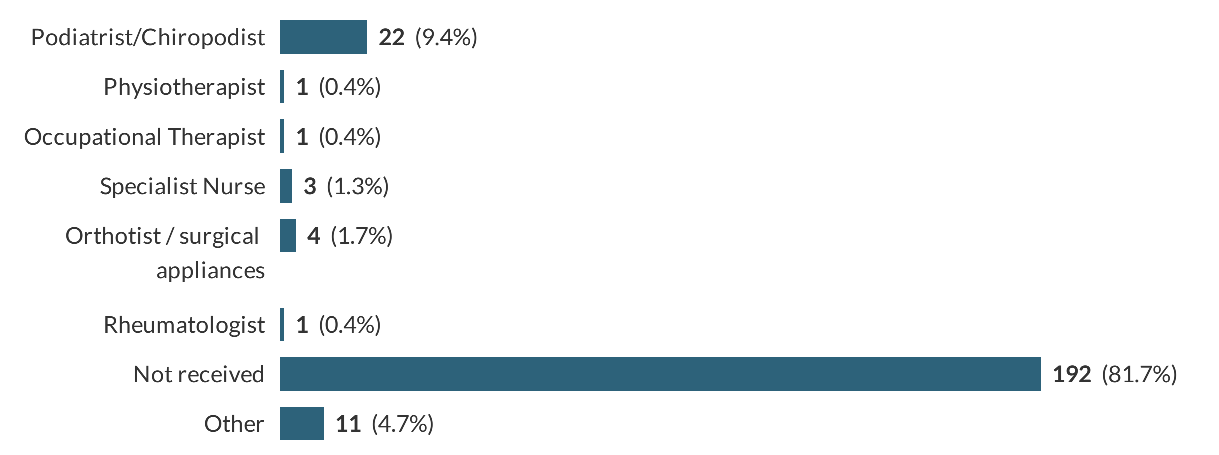

10.6.b Audiovisual aids such as pictures of footwear/insoles or images of feet - If you selected Other, please specify:

Showing 5 of 11 responses	
Arthritis charity website	120841-120835-6761354
NRAS meetings	120841-120835-6761434
Internet	120841-120835-6761450
NRAS info day about footcare	120841-120835-6761454
York rheumatoid arthritis support group	120841-120835-6761605

10.7 Been given information about websites such as NRAS, Arthritis Care, Arthritis Research UK

10.7.a Been given information about websites such as NRAS, Arthritis Care, Arthritis Research UK - Tick the boxes for each type of education you have received and indicate who you received it from according to the options in the right hand columns.

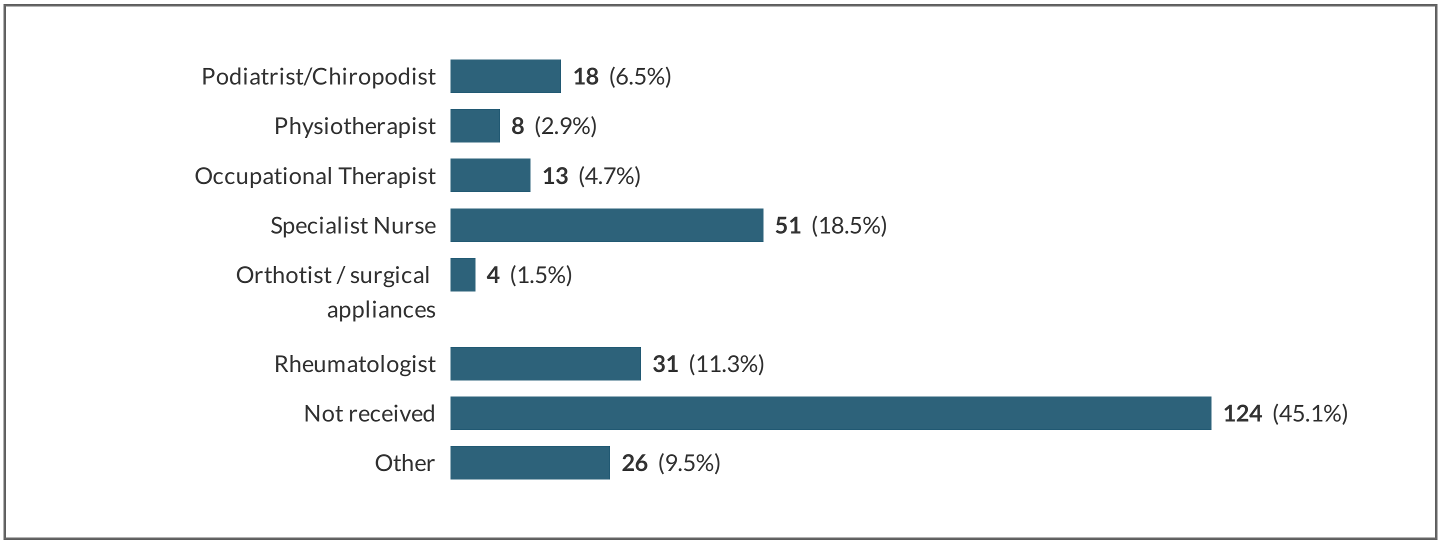

10.7.b Been given information about websites such as NRAS, Arthritis Care, Arthritis Research UK - If you selected Other, please specify:

**Showing 5 of 26 responses**	
Healthcare @ Home nurses who delivers my anti-TNFs	120841-120835-6761305
Internet search	120841-120835-6761314
self help	120841-120835-6761318
found it myself	120841-120835-6761346
From a friend	120841-120835-6761396

11 To what extent do you think these methods of education are effective, in relation to your foot health?

11.1 Verbal Information

11.1.a Verbal Information - Please select from Very Effective to Don't KNow

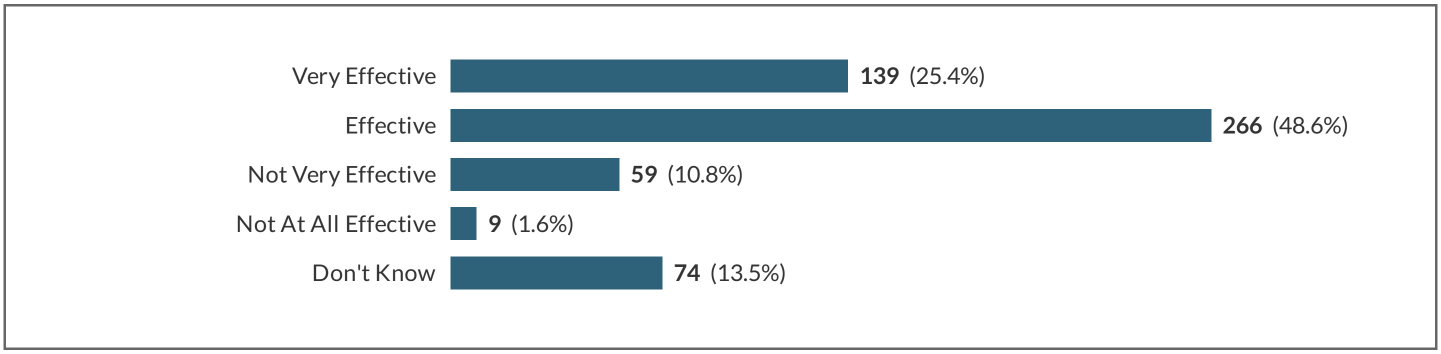

11.2 Written Information

11.2.a Written Information - Please select from Very Effective to Don't KNow

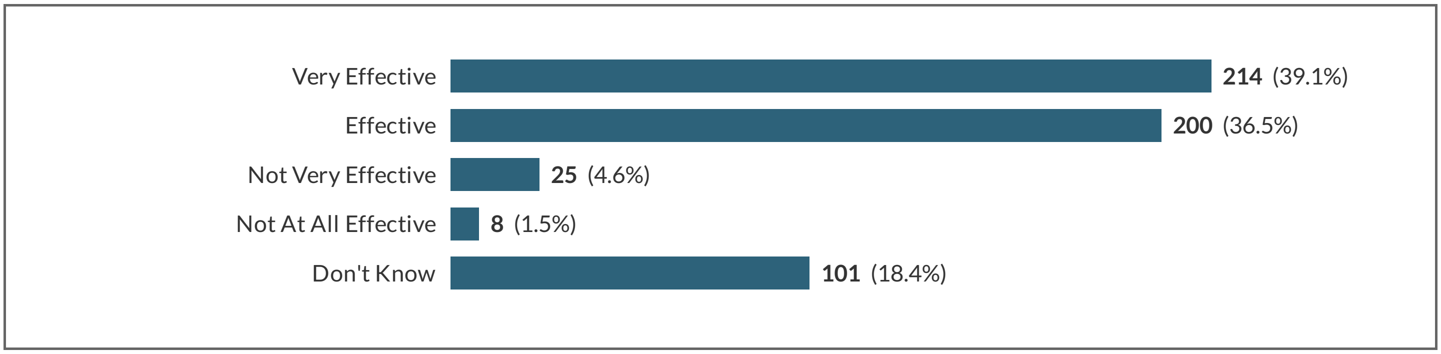

11.3 Group Education sessions

11.3.a Group Education sessions - Please select from Very Effective to Don't KNow

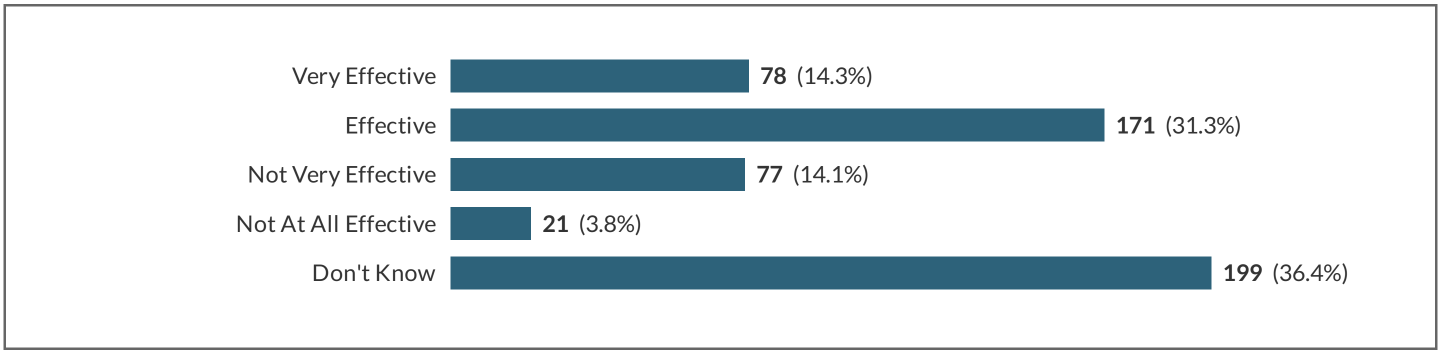

11.4 Audio-visual aids such as videos/demonstrations

11.4.a Audio-visual aids such as videos/demonstrations - Please select from Very Effective to Don't KNow

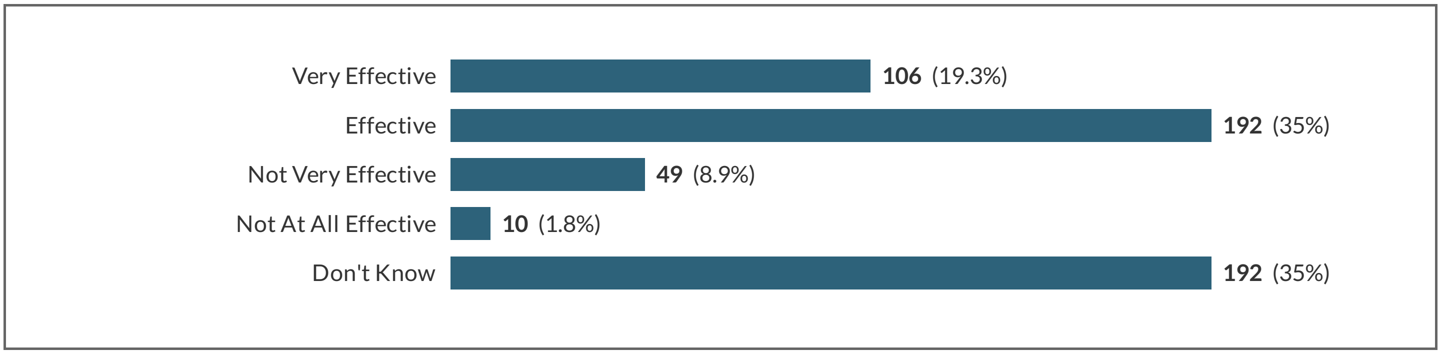

11.5 Web sites

11.5.a Web sites - Please select from Very Effective to Don't KNow

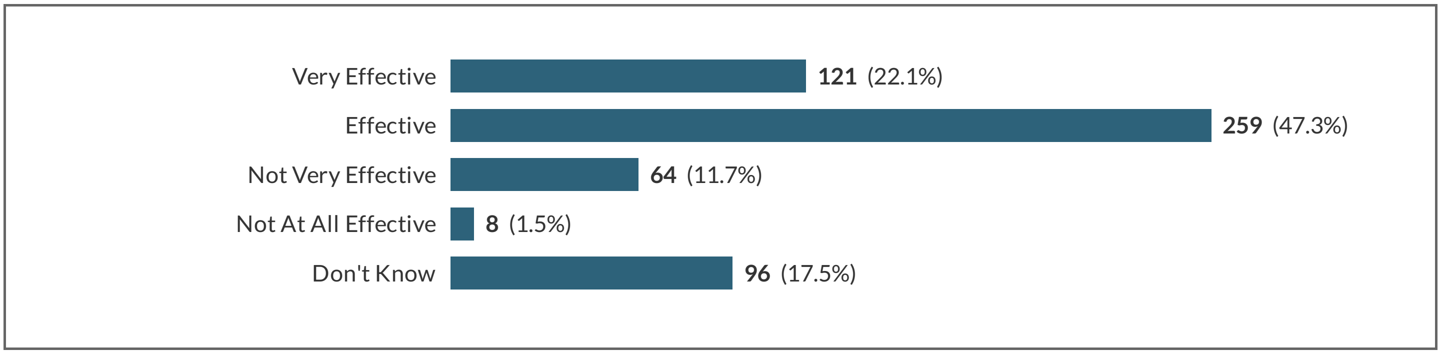

11.6 Other (in relation to additional methods you gave)

11.6.a Other (in relation to additional methods you gave) - Please select from Very Effective to Don't KNow

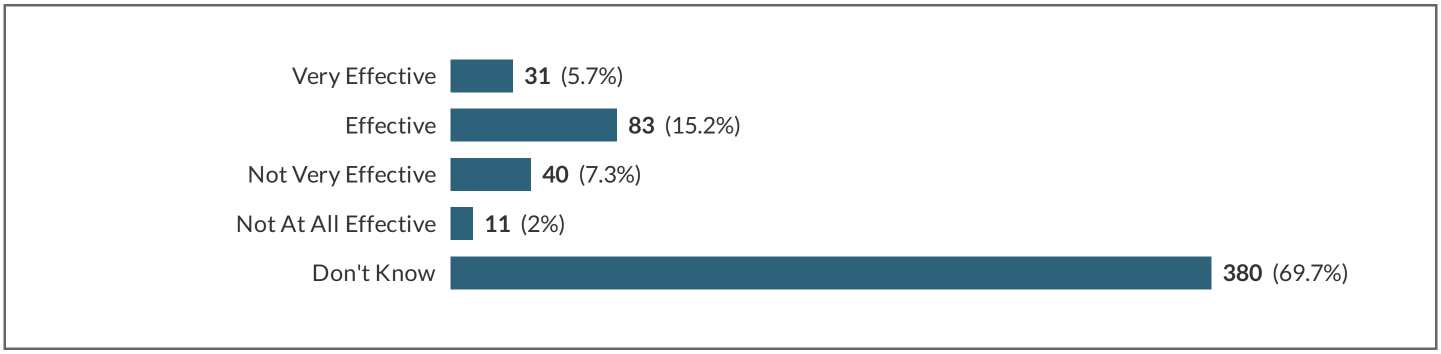

Section 4: What should be included in foot health education provision?

12 To what extent do you think it is important for people with RA to know about the following areas of foot health education? Select from Very Important to Not Important for each item. They are in no particular order.

12.1 The role of the podiatrist/chiropodist in looking after my foot health

12.1.a The role of the podiatrist/chiropodist in looking after my foot health - Please select from Very Important to Not Important

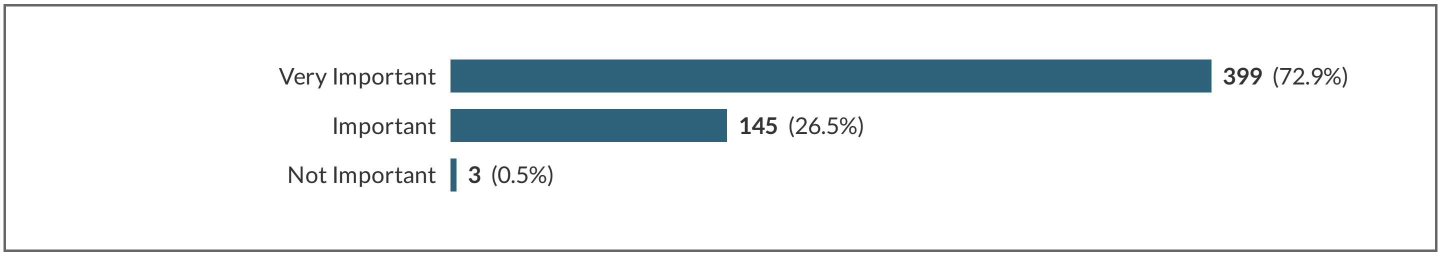

12.2 General disease related information (e.g what is RA? Causes etc..)

12.2.a General disease related information (e.g what is RA? Causes etc..) - Please select from Very Important to Not Important

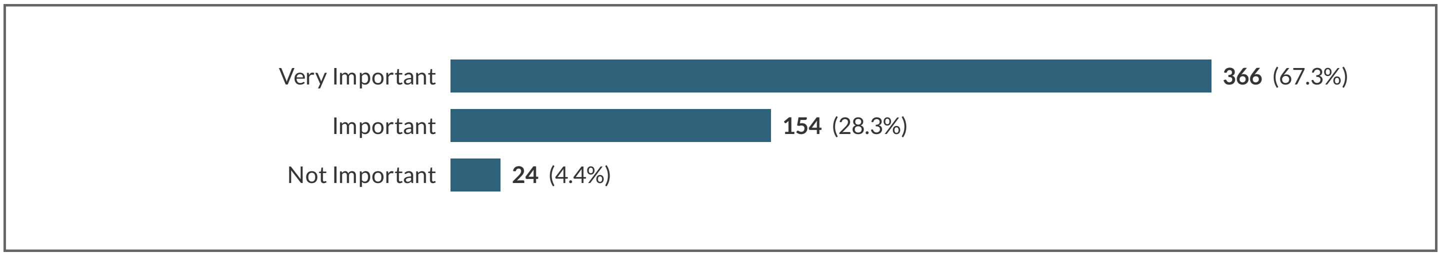

12.3 Information about how the medication I take for RA can affect my feet

12.3.a Information about how the medication I take for RA can affect my feet - Please select from Very Important to Not Important

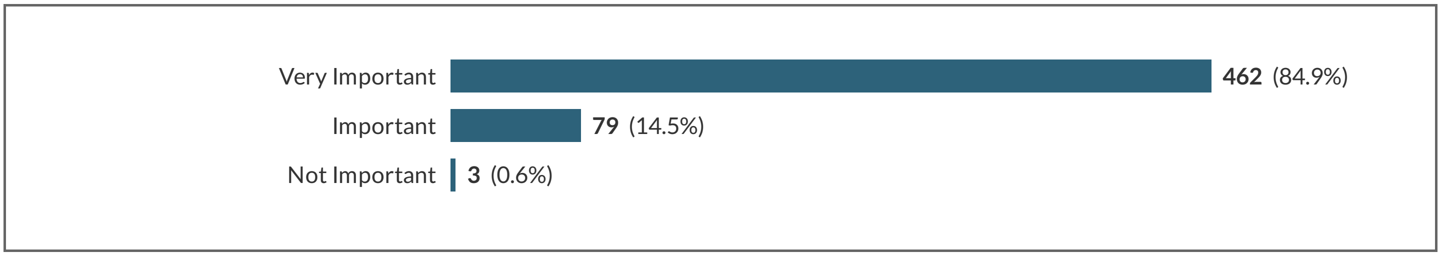

12.4 How RA affects the feet

12.4.a How RA affects the feet - Please select from Very Important to Not Important

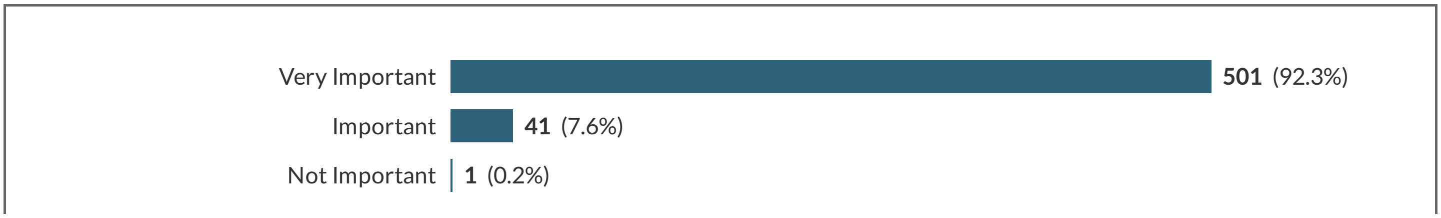

12.5 Contact details for podiatry/chiropody services (what is an emergency, how and who to contact)

12.5.a Contact details for podiatry/chiropody services (what is an emergency, how and who to contact) - Please select from Very Important to Not Important

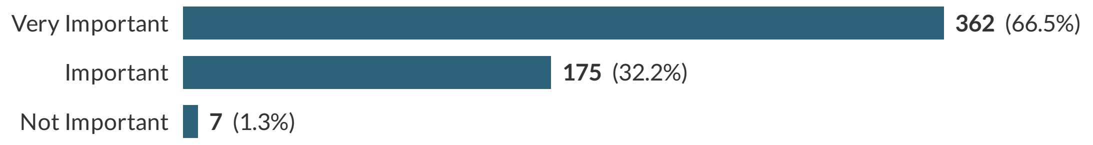

12.6 The different kinds of treatment I can have to help me with my foot problems

12.6.a The different kinds of treatment I can have to help me with my foot problems - Please select from Very Important to Not Important

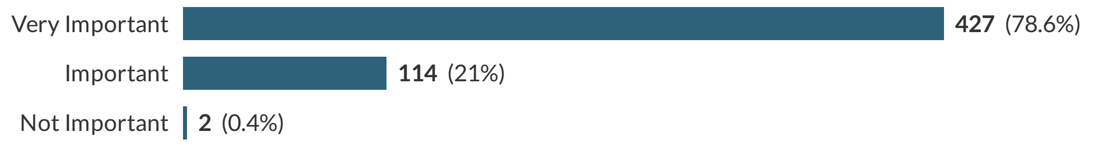

12.7 How to look after my own foot health (footwear advice, how to cut nails safely, use of moisturiser..)

12.7.a How to look after my own foot health (footwear advice, how to cut nails safely, use of moisturiser..) - Please select from Very Important to Not Important

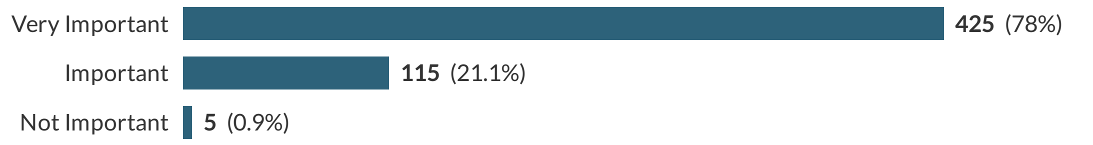

12.8 What might happen if I don't look after my feet

12.8.a What might happen if I don't look after my feet - Please select from Very Important to Not Important

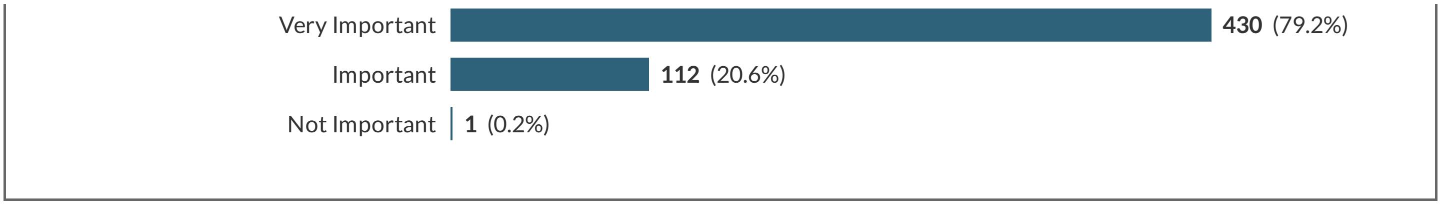

12.9 How other health professionals might be involved in looking after my feet.

12.9.a How other health professionals might be involved in looking after my feet. - Please select from Very Important to Not Important

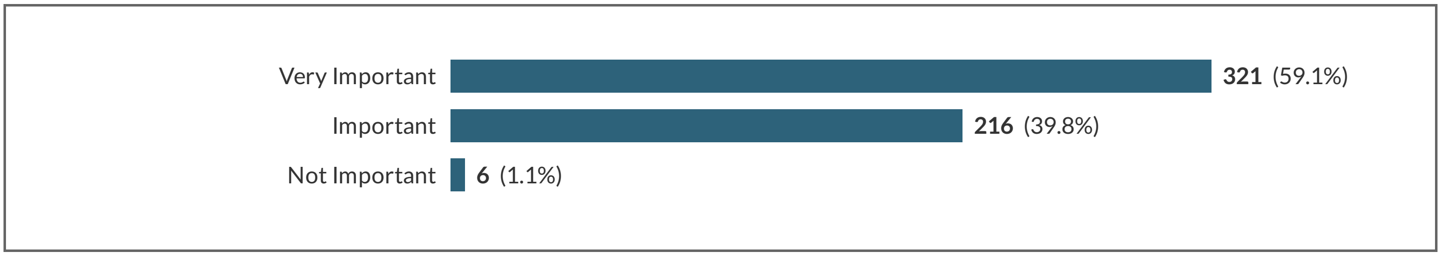

12.10 Information relating to Patient support groups/resources such as websites (e.g NRAS, Arthritis Care)

12.10.a Information relating to Patient support groups/resources such as websites (e.g NRAS, Arthritis Care) - Please select from Very Important to Not Important

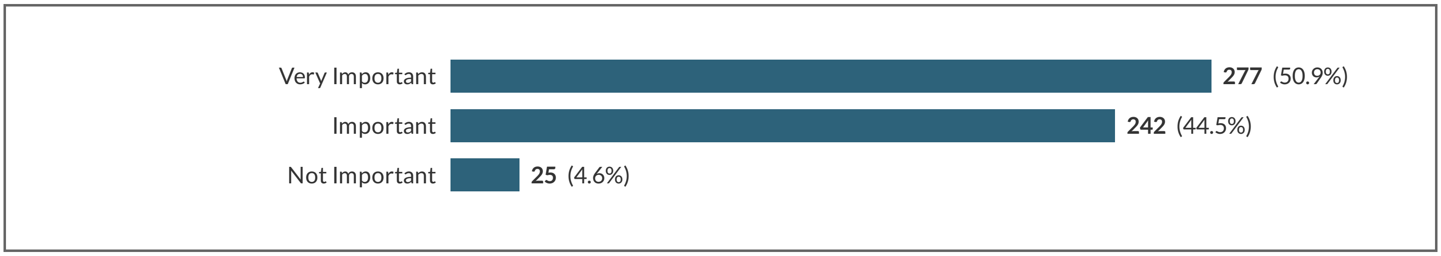

Section 5: When is the best time to receive foot health education?

13 When do you think is the best time for you to be given foot health education relating to RA? Select all that you think apply.

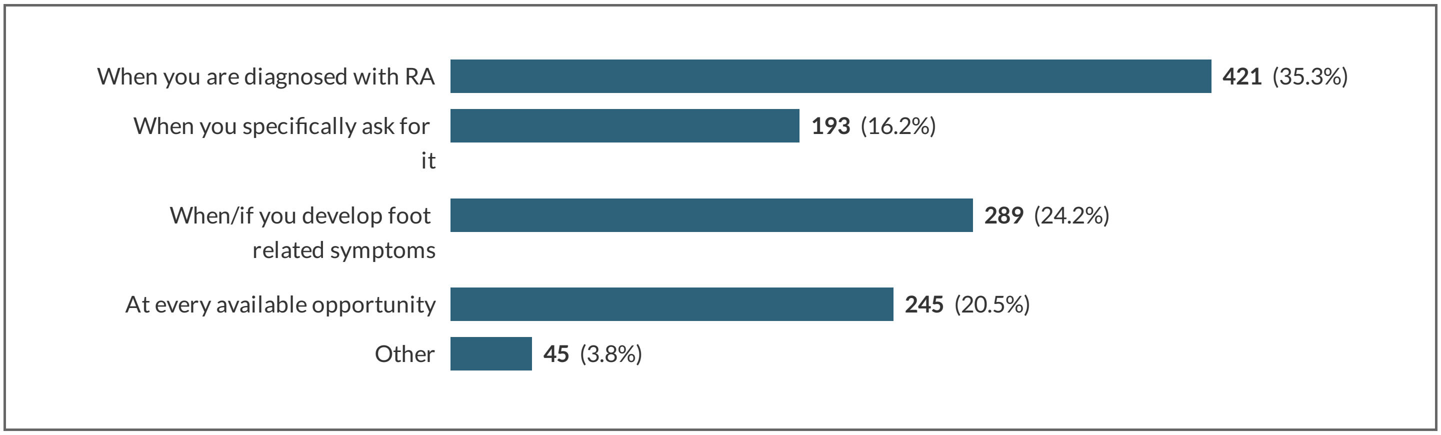

13.a If you selected Other, please specify:

Showing 5 of 45 responses	
I think the specialist nurses and consultants should take my foot pain much more seriously. I told them I had very painful lumps under my feet, they shrug and say nodules. But what are nodules, what can they do for me? No idea.	120841-120835-6761308
I now suffer from a limp from a badly managed Achilles tendon rupture. Never offered any supports which may have protected my ankle from the forementioned damage.	120841-120835-6761318
Perhaps automatically at annual review appointments.<br>There is a lot to take in at point of diagnosis, so perhaps again after 3 & 6 months initially.	120841-120835-6761321
it seems not taken into account	120841-120835-6761341
When the diagnosis has had time to sink in - there is so much to take in at the beginning, I think foot health education would not be appropriate then.	120841-120835-6761346

Section 6: Accessing foot health education

14 To what extent do you agree with the following statements? Select one response for each item.

14.1 I am clear about what questions to ask my podiatrist or other health professional regarding my foot health

14.1.a I am clear about what questions to ask my podiatrist or other health professional regarding my foot health - Please select from Strongly Agree to Strongly Disagree for each item. If you are unsure please select 'Don't Know'. There is space in the final column for each item if you wish to add any other comment.

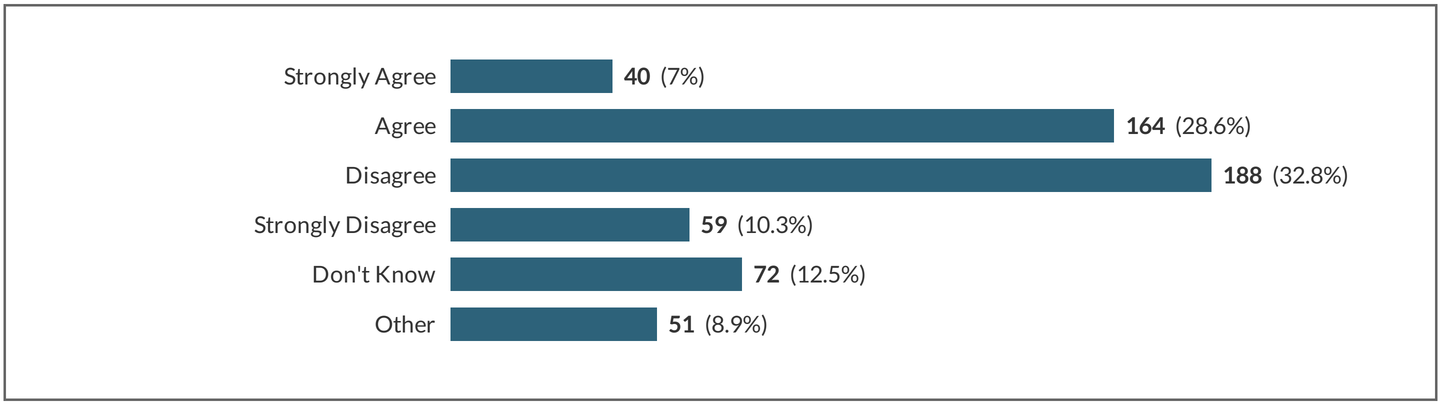

14.1.b I am clear about what questions to ask my podiatrist or other health professional regarding my foot health - If you selected Other, please specify:

Showing 5 of 51 responses	
Don't really know what the podiatrist does	120841-120835-6761308
Never offered services	120841-120835-6761318
Have never had an appointment	120841-120835-6761353
No offer of referral to podiatry. Never referred to at Rhumatology appointments. All they are interested in is x-raying my feet!	120841-120835-6761354
Not applicable at this time	120841-120835-6761363

14.2 There is enough time during my appointment with the chiropodist/podiatrist to ask questions about foot health education

14.2.a There is enough time during my appointment with the chiropodist/podiatrist to ask questions about foot health education - Please select from Strongly Agree to Strongly Disagree for each item. If you are unsure please select 'Don't Know'. There is space in the final column for each item if you wish to add any other comment.

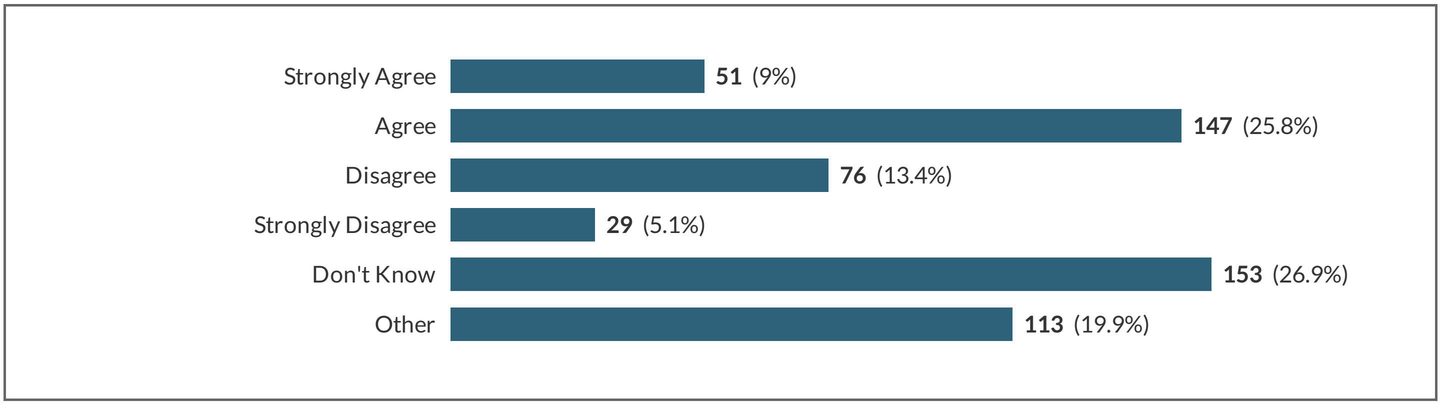

14.2.b There is enough time during my appointment with the chiropodist/podiatrist to ask questions about foot health education - If you selected Other, please specify:

Showing 5 of 113 responses	
I have no foot care offered	120841-120835-6761307
N/A have not been offered podiatry/chiropody support	120841-120835-6761309
I've never been offered an appointment	120841-120835-6761316
Not been	120841-120835-6761327
have not been offered podiatry care	120841-120835-6761331

14.3 My podiatrist/chiropodist asks about my foot health concerns during my appointment

14.3.a My podiatrist/chiropodist asks about my foot health concerns during my appointment - Please select from Strongly Agree to Strongly Disagree for each item. If you are unsure please select 'Don't Know'. There is space in the final column for each item if you wish to add any other comment.

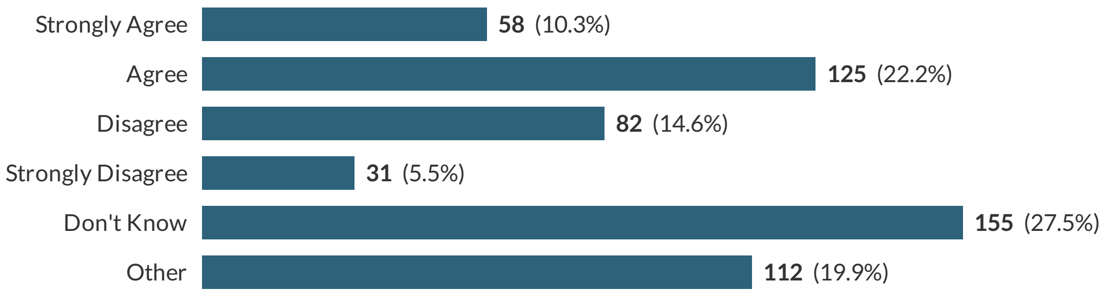

14.3.b My podiatrist/chiropodist asks about my foot health concerns during my appointment - If you selected Other, please specify:

Showing 5 of 112 responses	
I have no foot care offered	120841-120835-6761307
He won't do anything though as I am " in a flare" but I think that's exactly when I need help with my feet.	120841-120835-6761308
N/A have not been offered podiatry/chiropody support	120841-120835-6761309
I don't have one	120841-120835-6761316
Never offered services.	120841-120835-6761318

14.4 Other Health Professionals (such as the Specialist Nurse, Physio, Occupational Therapist or Consultant)ask about my feet during my appointment

14.4.a Other Health Professionals (such as the Specialist Nurse, Physio, Occupational Therapist or Consultant)ask about

my feet during my appointment - Please select from Strongly Agree to Strongly Disagree for each item. If you are unsure please select 'Don't Know'. There is space in the final column for each item if you wish to add any other comment.

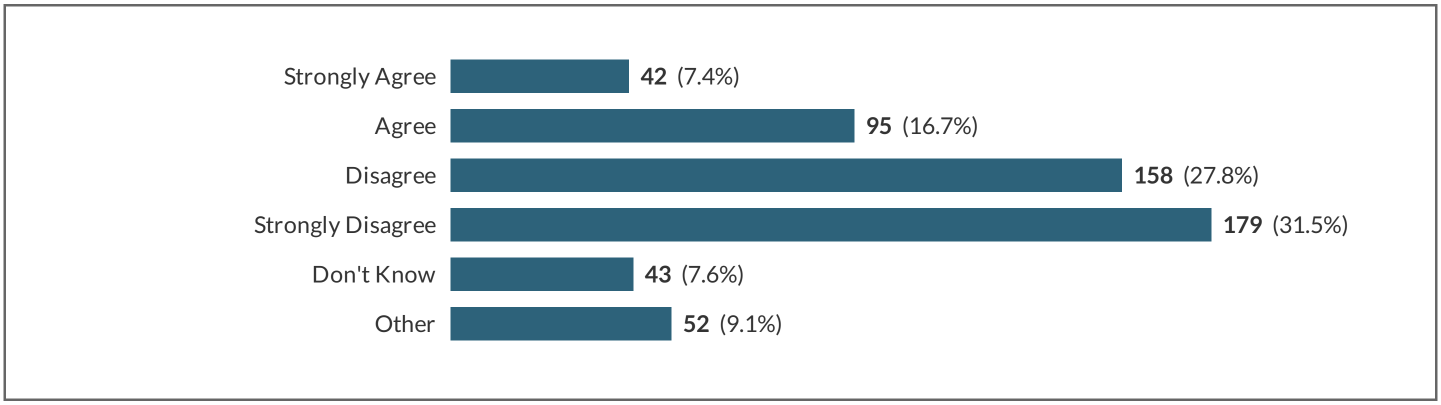

14.4.b Other Health Professionals (such as the Specialist Nurse, Physio, Occupational Therapist or Consultant)ask about my feet during my appointment - If you selected Other, please specify:

Showing 5 of 52 responses	
Only if I bring up my concerns re: foot health.	120841-120835-6761305
Really feel they aren't bothered as its not in the DAS score, therefore not a high priority	120841-120835-6761308
Recently spoke to a physio who advised on foot wear.	120841-120835-6761318
Occupational therapist sometimes asks but I was referred to her about my hands	120841-120835-6761336
wife askes nurse about my feet	120841-120835-6761360

14.5 I have received written foot health information from my Podiatrist or other Health Professional

14.5.a I have received written foot health information from my Podiatrist or other Health Professional - Please select from Strongly Agree to Strongly Disagree for each item. If you are unsure please select 'Don't Know'. There is space in the final column for each item if you wish to add any other comment.

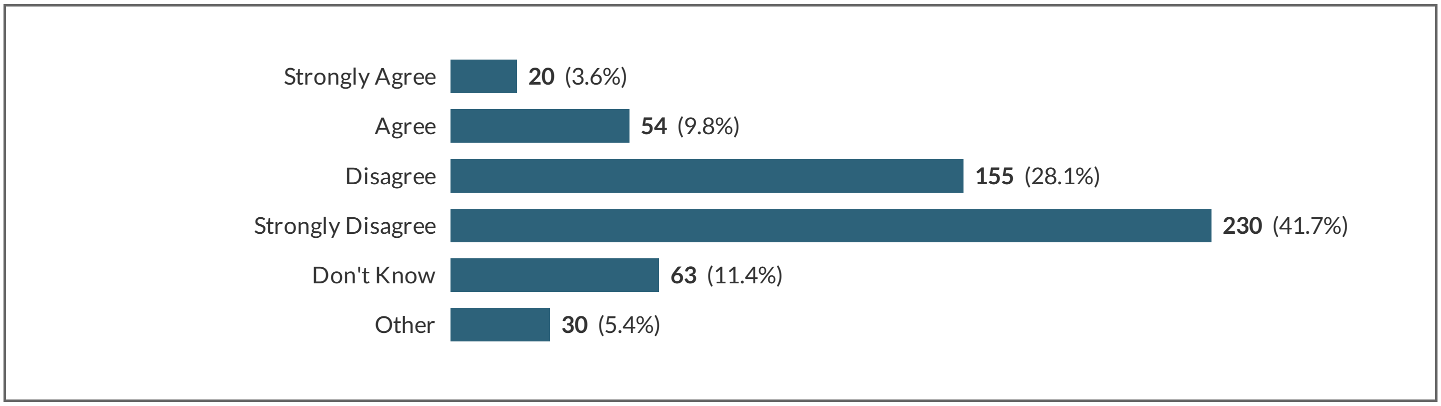

14.5.b I have received written foot health information from my Podiatrist or other Health Professional - If you selected

Other, please specify:

Showing 5 of 30 responses	
Not been	120841-120835-6761327
I never knew it exixted will start looking as my feet are very bad	120841-120835-6761353
only on inserts for my shoes	120841-120835-6761360
Not applicable at this time	120841-120835-6761363
no information received	120841-120835-6761435

14.6 I have received or found information myself relating to RA and foot health, but found it difficult to understand

14.6.a I have received or found information myself relating to RA and foot health, but found it difficult to understand - Please select from Strongly Agree to Strongly Disagree for each item. If you are unsure please select 'Don't Know'. There is space in the final column for each item if you wish to add any other comment.

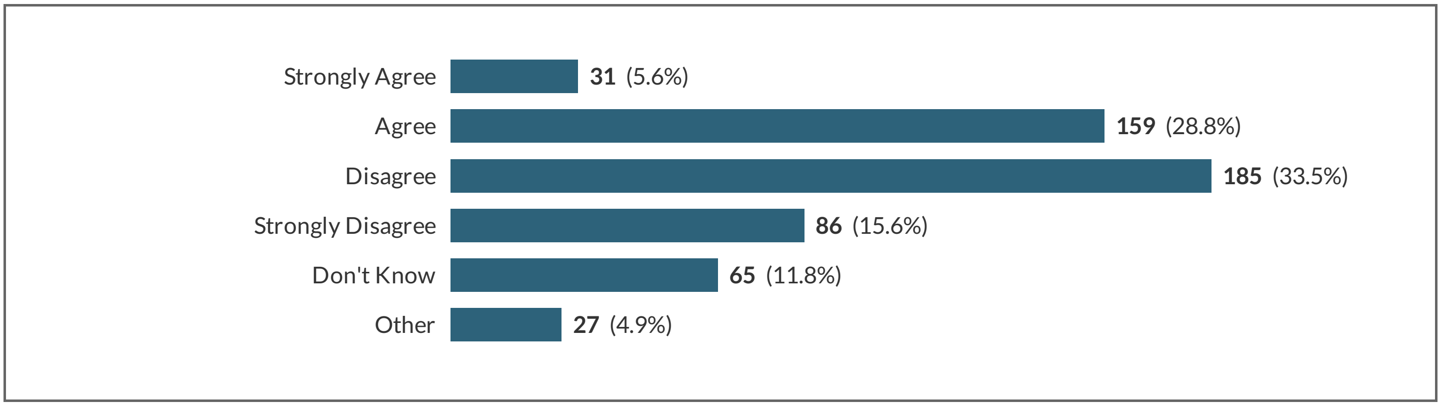

14.6.b I have received or found information myself relating to RA and foot health, but found it difficult to understand - If you selected Other, please specify:

Showing 5 of 27 responses	
This is the first I've heard of it	120841-120835-6761316
NRAS information	120841-120835-6761321
I found the info myself & Lund it easy to understand, but I am a Nurse Educator with RA.	120841-120835-6761354
Didn't know there was any. Didn't know I should be concerned about my feet.	120841-120835-6761378
I sent for info about foot surgery from bras, which was interesting and not particularly hard to understand it was however, rather out if date	120841-120835-6761396

14.7 I know where I can access written foot health information (such as a leaflet or from websites such as NRAS)

14.7.a I know where I can access written foot health information (such as a leaflet or from websites such as NRAS) - Please select from Strongly Agree to Strongly Disagree for each item. If you are unsure please select 'Don't Know'. There is space in the final column for each item if you wish to add any other comment.

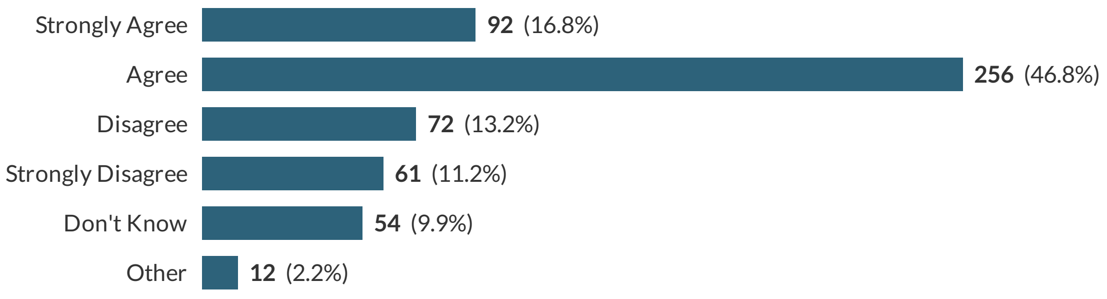

14.7.b I know where I can access written foot health information (such as a leaflet or from websites such as NRAS) - If you selected Other, please specify:

Showing 5 of 12 responses	
one would only think about foot health information and how to go about it once there was a problem, first stop would probably be GP	120841-120835-6761419
Need to get someone to access information for me.	120841-120835-6761458
I know where the local Poidiatrist clinics are held.	120841-120835-6761476
As above.	120841-120835-6761379
As above	120841-120835-6761549

14.8 I am able to access the Internet or someone does it for me

14.8.a I am able to access the Internet or someone does it for me - Please select from Strongly Agree to Strongly Disagree for each item. If you are unsure please select 'Don't Know'. There is space in the final column for each item if you wish to add any other comment.

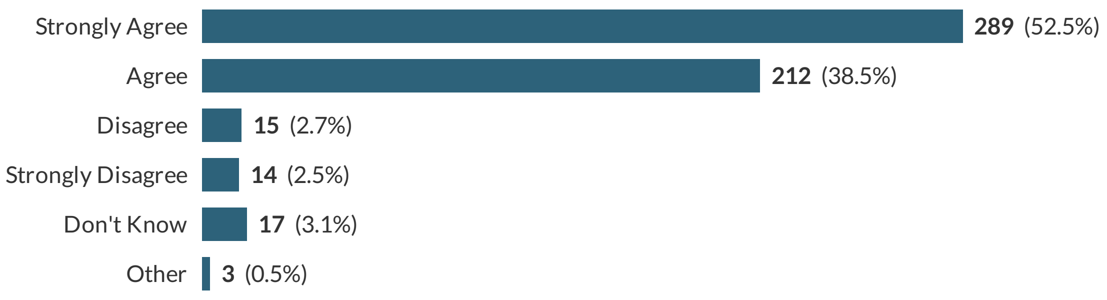

14.8.b I am able to access the Internet or someone does it for me - If you selected Other, please specify:

Showing all 3 responses	
Yes have access to Internet.	120841-120835-6761379
As above	120841-120835-6761549
Myself.	120841-120835-6761764

14.9 I cannot afford the time to attend patient support group meetings

14.9.a I cannot afford the time to attend patient support group meetings - Please select from Strongly Agree to Strongly Disagree for each item. If you are unsure please select 'Don't Know'. There is space in the final column for each item if you wish to add any other comment.

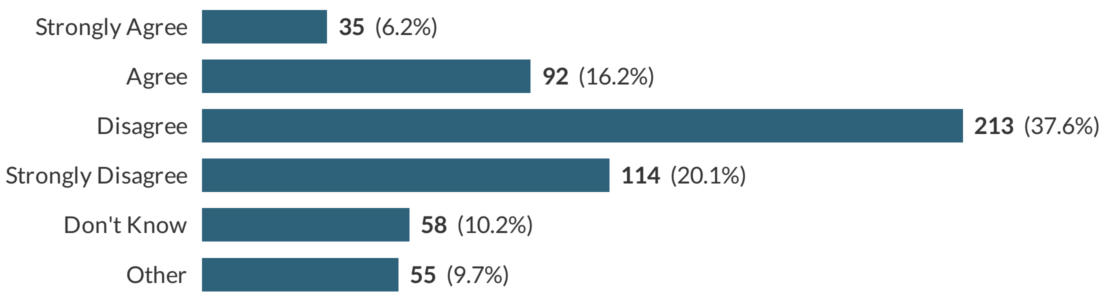

14.9.b I cannot afford the time to attend patient support group meetings - If you selected Other, please specify:

Showing 5 of 55 responses	
Also it's very painful to get to meetings due to sore joints and painful feet. The rheumatology department is at the back of the hospital too.	120841-120835-6761308
The times are what prevent me from attending	120841-120835-6761310
I can now since taking voluntary retirement.	120841-120835-6761318
I cannot attend on my own, so it would involve another person's time.	120841-120835-6761321
not been offered these	120841-120835-6761341

14.10 I cannot afford the money to attend patient support group meetings

14.10.a I cannot afford the money to attend patient support group meetings - Please select from Strongly Agree to Strongly Disagree for each item. If you are unsure please select 'Don't Know'. There is space in the final column for each item if you wish to add any other comment.

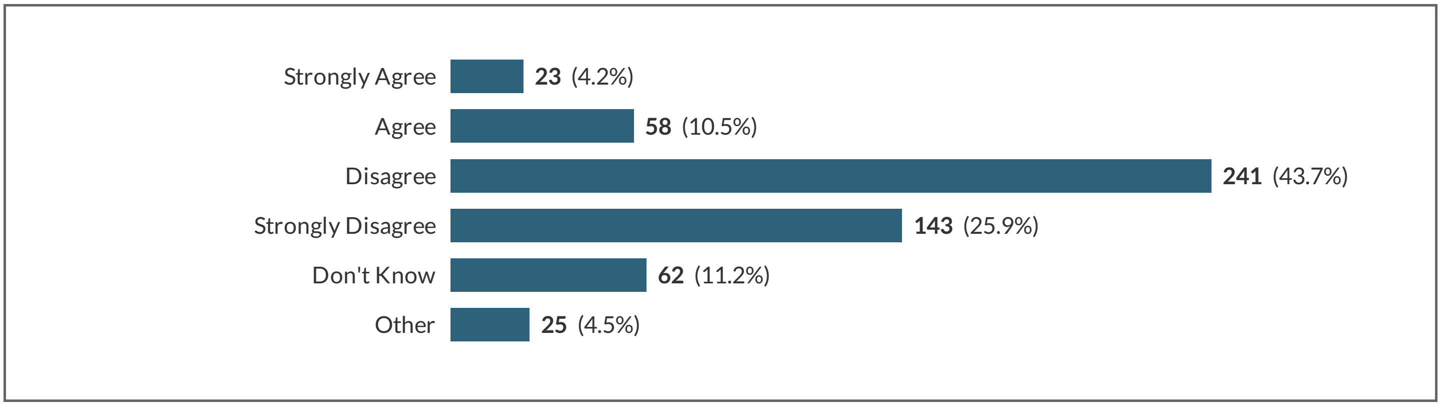

14.10.b I cannot afford the money to attend patient support group meetings - If you selected Other, please specify:

**Showing 5 of 25 responses**	
There are no patient support groups here that I can access - all require a car and I don't have one!	120841-120835-6761326
as above	120841-120835-6761341
see above	120841-120835-6761346
I don't know if there are any?	120841-120835-6761377
doesn't cost money	120841-120835-6761404

15 If you use the internet to get foothealth information, which websites do you most frequently use? please select all that apply and add any other website that we may not have included in the 'other' box.

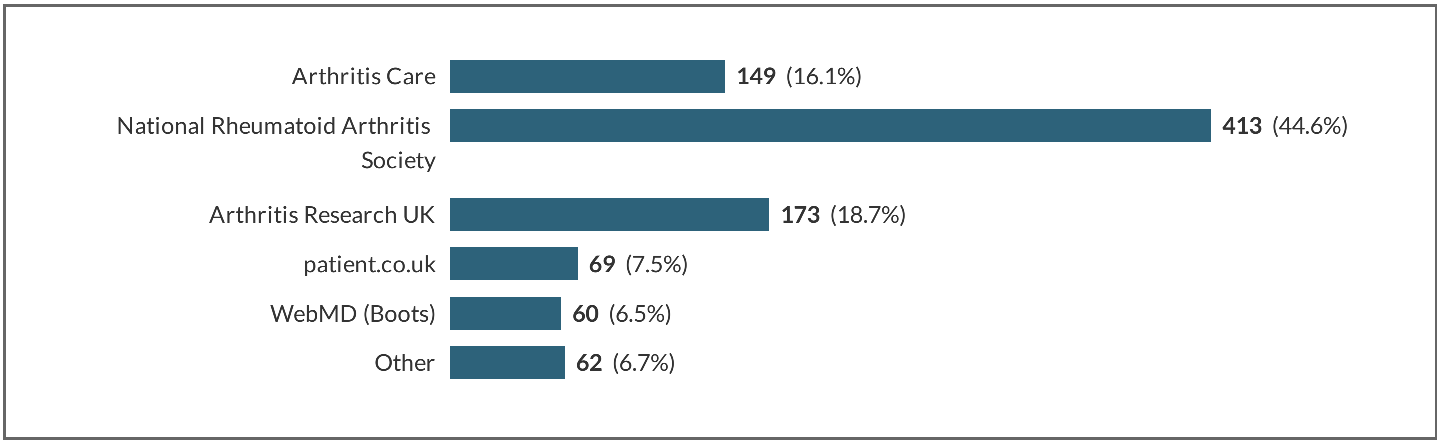

15.a If you selected Other, please specify:

Showing 5 of 62 responses	
I Google my problem and see what comes up.	120841-120835-6761312
I didn't know that you had to take care of your feet. With my feet changing shape it would of been good to have known	120841-120835-6761316
Amazon for specialist products.	120841-120835-6761318
Health Unlocked	120841-120835-6761326
I try any and all websites although I find the US ones a bit sensational.	120841-120835-6761335

16 This free text box is for you to add any additional comments or information that you feel is relevant and has not been addressed by this survey. Thank you.

Showing 5 of 249 responses	
I have had a letter today from my GP offering this servce	120841-120835-6761307
I have extremely painful feet and no one takes any notice of my feet pain as it isn't included in my DAS score, so they are not bothered. But for my pain and quality of life it is the biggest thing! Also I was give a pair of thin insoles by a friend hitch are pretty useless.	120841-120835-6761308
Question 11 is ambiguous (as far as I understand) if Question 10 doesn't apply. Particularly Question 11f but the survey says these questions are mandatory. This seems to be a confusing and poorly designed survey for the user.	120841-120835-6761309
Feet are of little interest to the RA professionals. My feet seem to cause me a lot of trouble, but there is no obvious person to ask.	120841-120835-6761312
I don't think enough information/ advice is given during appointments (my experience only) and I have always had to raise the subject myself.	120841-120835-6761314
