# Supplementary material for: A survey of people with foot problems related to rheumatoid arthritis and their educational needs
Source: J Foot Ankle Res. 2017 Mar 6;10:12. doi: 10.1186/s13047-017-0193-6 (PMC5340002; doi:10.1186/s13047-017-0193-6)
Supplement: Additional file 2: — P-values arising from statistical analyses of participants’ survey responses by section, in relation to gender, age, disease duration and living situation. * - Denotes significance at the 5% level. (DOCX 80 kb) [file 13047_2017_193_MOESM2_ESM.docx]

Additional file 2: P-values arising from statistical analyses of participants’ survey responses by section, in relation to gender, age, disease duration and living situation. * - Denotes significance at the 5% level.

| **Independent Variables** | | | | |
| --- | --- | --- | --- | --- |
| **Dependent Variables: by FHE survey section** | Gender | Age | Disease duration | Living situation |
| Aims | 0.504 | 0.073 | 0.279 | 0.187 |
| Methods: written | 0.877 | 0.132 | 0.409 | 0.008* |
| Methods: Verbal | 0.329 | 0.225 | 0.140 | 0.228 |
| Methods: Group | 0.284 | 0.467 | 0.878 | 0.519 |
| Methods: A-V videos | 0.350 | 0.521 | 0.462 | 0.805 |
| Methods: A-V demos | 0.589 | 0.929 | 0.398 | 0.277 |
| Methods: Images | 0.464 | 0.254 | 0.354 | 0.354 |
| Methods: websites | 0.245 | 0.204 | 0.090 | 0.737 |
| Effectiveness of methods | 0.150 | 0.720 | 0.842 | 0.899 |
| Content | 0.022* | 0.886 | 0.956 | 0.144 |
| Timing | 0.019* | 0.106 | 0.163 | 0.894 |
| Barriers (+ve statements) | 0.527 | 0.004* | 0.241 | 0.985 |
| Barriers (-ve statements) | 0.547 | 0.535 | 0.147 | 0.876 |
| Web sites used | 0.034* | 0.146 | 0.342 | 0.498 |
